# A High-Density Simple Sequence Repeat-Based Genetic Linkage Map of Switchgrass

**DOI:** 10.1534/g3.111.001503

**Published:** 2012-03-01

**Authors:** Linglong Liu, Yanqi Wu, Yunwen Wang, Tim Samuels

**Affiliations:** Department of Plant and Soil Sciences, Oklahoma State University, Stillwater, Oklahoma 74078

**Keywords:** linkage map, simple sequence repeat (SSR), selfed progeny, switchgrass

## Abstract

Switchgrass (*Panicum virgatum*) has been identified as a promising cellulosic biofuel crop in the United States. Construction of a genetic linkage map is fundamental for switchgrass molecular breeding and the elucidation of its genetic mechanisms for economically important traits. In this study, a novel population consisting of 139 selfed progeny of a northern lowland genotype, NL 94 LYE 16X13, was used to construct a linkage map. A total of 2493 simple sequence repeat markers were screened for polymorphism. Of 506 polymorphic loci, 80.8% showed a goodness-of-fit of 1:2:1 segregation ratio. Among 469 linked loci on the framework map, 241 coupling *vs.* 228 repulsion phase linkages were detected that conformed to a 1:1 ratio, confirming disomic inheritance. A total of 499 loci were mapped to 18 linkage groups (LG), of which the cumulative length was 2085.2 cM, with an average marker interval of 4.2 cM. Nine homeologous LG pairs were identified based on multi-allele markers and comparative genomic analysis. Two clusters of segregation-distorted loci were identified on LG 5b and 9b, respectively. Comparative analysis indicated a one-to-one relationship between nine switchgrass homeologous groups and nine foxtail millet (*Setaria italica*) chromosomes, suggesting strong homology between the two species. The linkage map derived from selfing a heterozygous parent, instead of two separate maps usually constructed for a cross-fertilized species, provides a new genetic framework to facilitate genomics research, quantitative trait locus (QTL) mapping, and marker-assisted breeding.

Switchgrass (*Panicum virgatum* L.) is one of the dominant C4 perennial species present in the North American tall grass prairies. Its natural habitat extends to a larger geographic span between about 15 and 55 degree north latitudes (Hitchcock 1951). According to gross morphology and habitat preference, switchgrass is classified mainly into lowland and upland ecotypes (Porter 1966). Lowland plants are tetraploid (2n = 4x = 36 chromosomes), whereas uplands include both tetraploid and octoploid plants (2n = 8x = 72) (Hopkins *et al.* 1996). Aneuploidy is common in both lowland and upland plants, although octoploid upland plants have more aneuploidy incidences than tetraploid accessions (Costich *et al.* 2010). Molecular marker investigations have revealed enormous genetic diversity within the species (Gunter *et al.* 1996; Narasimhamoorthy *et al.* 2008; Zalapa *et al.* 2011; Zhang *et al.* 2011).

Switchgrass is a tall growing and resilient species. Its genetic diversity has historically been used for soil conservation, forage production, game cover, and as an ornamental grass. More recently, it has been selected as the model herbaceous species for use as a dedicated bioenergy feedstock crop (McLaughlin and Kszos 2005). Switchgrass is listed as one of the major biomass energy crops in the Billion-Ton Update report (U.S. Department of Energy 2011). In a farm-scale study of switchgrass grown as a biomass energy crop on marginal cropland, Schmer *et al.* (2008) reported switchgrass produces 540% more energy than the energy used for producing its cellulosic feedstock. They estimated greenhouse gas emissions from converting switchgrass feedstock to ethanol were 94% lower than that from gasoline. Switchgrass has received substantial attention and has the potential to be genetically improved for higher biomass production along with other important agronomic traits that can add value to its use as a biofuel feedstock in breeding programs.

Switchgrass is a wind pollinated and largely self-incompatible species (Talbert *et al.* 1983; Taliaferro *et al.* 1999; Martinez-Reyna and Vogel 2002). Because of this sexually out-crossing mode of reproduction, all released cultivars were populations composed of genetically heterozygous individuals. Recently released switchgrass cultivars were primarily developed using recurrent selection procedures (Vogel *et al.* 2011). Those breeding and selection protocols are effective but require a lengthy time period to develop new cultivars. Consequently, genetic gains per year are relatively low (Vogel and Pedersen 1993). Molecular tools and genomic information are limited in switchgrass and need to be developed. These new and quickly evolving technologies have extensive potential if incorporated into and coupled with conventional genetic improvement and breeding programs for developing superior cultivars.

Molecular markers have been developed to investigate inheritance in the species and facilitate the construction of genetic linkage maps. These maps are fundamental for switchgrass breeding through marker-assisted selection and elucidation of the genetic mechanisms for economically important traits. The first linkage maps were constructed with 102 restriction fragment length polymorphism (RFLP) single dosage markers (Missaoui *et al.* 2005). The markers are distributed in eight homology groups covering over 400 cM. Developing microsatellites or simple sequence repeat (SSR) markers, which are tandem repeats of short (1 to 6 bp) DNA sequences, has gained substantial attention in switchgrass (Tobias *et al.* 2005, 2008; Wang *et al.* 2011). The desirable features of SSR markers include their easy use, high information content, codominant inheritance pattern, even distribution along chromosomes, reproducibility, and locus specificity (Kashi *et al.* 1997; Röder *et al.* 1998a,b). A pair of genetic maps using SSRs scored as single dosage markers has been developed in switchgrass (Okada *et al.* 2010). These maps covered, respectively, 1376 and 1645 cM of 18 linkage groups that are expected to represent the full set for a tetraploid genome. Okada *et al.* (2010) reported that the two tetraploid switchgrass parents had complete or near-complete disomic inheritance.

Marker-assisted selection is more efficient when molecular maps are well saturated, as high-density maps provide increased opportunities for detecting polymorphic markers in genomic regions of interest. Linkage maps developed using different genetic backgrounds are needed to better understand inheritance in the species. Linkage maps constructed from different populations will enhance the understanding of the genome structure and gene interaction (Semagn *et al.* 2010). Two full-sib F1 populations were used to construct previously published maps (Missaoui *et al.* 2005; Okada *et al.* 2010). The F1 full-sib populations were made by crossing two selected heterozygous parental plants.

NL94 LYE 16x13 (abbreviated as NL94) is a self-compatible genotype selected from a breeding nursery of the OSU northern lowland population formed using Kanlow and other germplasm (Liu and Wu 2011). Selfing NL94 and other self-compatible lowland plants can produce promising inbred lines that offer numerous advantages in breeding, especially for utilizing heterosis by crossing selected heterotic inbreds. We have developed an inbred population from NL94 (Liu and Wu 2011). Accordingly, the major objective of this study was to construct a more saturated SSR-based linkage map using the inbred progeny population. This map would provide a new genetic framework to study associations of molecular markers and agronomic traits of interest.

## MATERIALS AND METHODS

### Plant materials and the selfed mapping population

The mapping population was recently described by Liu and Wu (2011). Basically, the mapping population consisted of 139 individuals randomly selected from 279 inbreds derived by selfing NL94, which was identified as a typical tetraploid lowland ecotype (2n = 4X = 36) based on the detection of the 49 bp deletion in *trnL*-*UAA* intron (see supporting information, Figure S1), a special marker for switchgrass classification between lowland and upland (Missaoui *et al.* 2006). The decision to use 139 progeny was dictated by our genotype-detecting equipment, which enables the organization of the entire mapping population in two 66-well plates plus a small marker screening panel including seven individuals and the parent.

### DNA isolation and PCR amplification

The genomic DNA for NL94 and its progeny plants was respectively isolated from healthy leaf tissues using the CTAB method (Doyle and Doyle 1990), with minor modifications as described by Liu and Wu (2011). To avoid allele dropout due to poor DNA quality, DNA samples with smear bands of A260/A280 less than 1.8 were extracted *de novo*. The working solutions were diluted to 10 ng/μl as PCR templates.

SSR markers were amplified using selected primer pairs (PP; described in the next section) on Biosystems 2720 thermal cyclers (Applied Biosystems, CA), using the PCR reaction conditions as described by Wang *et al.* (2011). PCR products were separated using 6.5% KB plus polyacrylamide gel solution on a LI-COR 4300 DNA Analyzer (LI-COR Biosciences, Lincoln, NE). The band sizes of these amplified fragments of SSR markers were determined using Saga Generation 2 software, version 3.3 (LI-COR Biosciences).

### SSR markers and genotyping analysis

A total of 2288 switchgrass SSR primer pairs were assembled from previous publications (Tobias *et al.* 2006, 2008; Okada *et al.* 2010; Wang *et al.* 2011). They were compared to each other using a specialized blast program called bl2seq in NCBI (www.ncbi.nlm.nih.gov/BLAST/) designed to exclude redundancy. Non-redundant markers were then selected for polymorphism. In addition, 354 sorghum (*Sorghum bicolor*) SSRs from Wu and Huang (2006) were tested for their transferability in switchgrass varieties “Cave-in-rock” and “Alamo.” Of 189 foxtail millet (*Setaria italica*) nonredundant SSRs, 80 were taken from Jia *et al.* (2009) (a primer pair “b255” was excluded from their primer list due to the same primer sequences with “b225”), and the remaining 109 were kindly provided by Dr. A. Doust (Botany Department, Oklahoma State University).

The markers were initially screened for informative segregation using a small screening panel. Polymorphic SSRs were used to genotype the first DNA panel of 66 individuals, and then those having stable, heritable, and reproducible markers were genotyped on the second panel of other 66 individuals. At last, the information from the small screening panel and the two 66-well panels was collected to represent the entire mapping population. The markers with greater than 10% missing data were genotyped again from those samples that did not have data in the previous genotyping runs.

### Marker scoring

All codominant markers were scored using the same segregation pattern (<hkxhk>: locus heterozygous in the parent, two alleles). SSR-amplified fragments were encoded as “hh” (only one upper band), “hk” (two bands), and “kk” (only one lower band). For dominant loci, “h-” was scored for presence, and “–” for absence. In both scenarios, “u” was recorded as missing data; this included unclear or ambiguous bands. If a marker produced multiple bands with the same segregation profile but with different sizes, only two main bands were recorded as segregating alleles and the other bands were omitted as redundant information. However, in addition to these main bands, if those markers produced stable secondary bands with different segregation profiles from the main bands, *i.e.* multi-allele markers, they were separately encoded by primer name, and the band size in the base pairs was used as a suffix for differentiating between them. All gel bands were manually scored by two independent people. Raw genotyping data are given in File S1.

### Segregation and linkage analysis

Several segregation ratios are possible in the selfed progeny derived from a tetraploid plant with two segregating bands (Table S1). The goodness-of-fit between observed and expected Mendelian ratios was analyzed for each marker locus using a χ^2^ test built in JoinMap 4.0 (Van Ooijen 2006). Markers that deviated from the theoretical expected ratios were considered distorted and were marked to indicate different significance levels (^*^P < 0.01, ^**^P < 0.001, and ^***^P < 0.0001).

Linkage analysis was performed using JoinMap 4.0, and the outcross pollinated (CP) full-sib family was used as the population type, which enabled the analysis of a self-pollinated population derived from a heterozygous parent. The linkage map was constructed in two steps. Initially, loci were grouped into linkage groups using the following parameters: the independence test log-likelihood of the odds (LOD) score ≥ 8.0, maximum-likelihood (ML) mapping module (Stam 1993; Jansen *et al.* 2001), Kosambi’s mapping function (Kosambi 1944), maximum recombination (REC) frequency = 0.35, goodness-of-fit Jump threshold for removal loci = 5.0, ripple = 1, and third round = yes. After grouping, loci within linkage groups were ordered using the regression mapping algorithm (Stam 1993). The linkage groups established from the third round of analysis formed the initial framework map. Then four to six loci distributed evenly on the framework map were fixed and the calculation parameter was changed to a LOD score ≥ 3.0 and a maximum REC frequency = 0.40. This step allowed us to assign some of the otherwise ungrouped loci on the already established linkage groups. Two independent linkage groups were accepted as linked if a marker on the end of one group showed a cross linkage to another marker on a second group through the “*maximum linkages*” function of JoinMap 4.0. In addition, those unmapped markers that showed weak links with mapped loci at the maximum linkage parameter threshold of 2.0 were listed next to mapped loci as accessory loci and formed the final linkage map. Markers showing segregation distortion were included in the final map if their presence did not alter surrounding marker order in a given linkage group. For any markers with an estimated position of less than 0 cM, their position was set as 0 cM, and the positions of other markers on the same linkage group were adjusted accordingly (Beldade *et al.* 2009). To compare the collinearity between the initial and final maps, the same markers and their individual distances on both maps were arrayed in Microsoft Excel 2007, and a function “correlate” was conducted to obtain a correlation coefficient.

Linkage groups (LG) were identified to be homeologous if they shared common SSR markers. Linkage groups were numbered based on the comparison with published linkage maps (Okada *et al.* 2010). The designation of subgenome “a” or “b” for each LG in this study was given according to the named subgenome of a corresponding LG, which shared more markers than its alternate LG (Okada *et al.* 2010). For a LG (*i.e.* 7b), on which only four gSSR markers mapped and no bridge markers were found, the original clone sequences harboring the four gSSR markers (Wang *et al.* 2011) were blasted against sorghum genome in GRAMENE (http://www.gramene.org/) with “near-exact matches” set as the search sensitivity parameter. Thus, using the sorghum genome sequence as a tool, LG 7b was identified and compared with the reference switchgrass maps of Okada *et al.* (2010).

The linkage phase of each locus on the final framework map was obtained from JoinMap 4.0, which automatically determined the coupling and repulsion phase types during the estimation of the recombination frequencies. Chi-square testing for the ratio of coupling to repulsion linkage phase was conducted with the online software of Preacher (2001).

### Comparative mapping

To search for the locations of the mapped SSR loci on foxtail millet chromosomes, foxtail millet genome sequence Phytozome v7.0 (http://www.phytozome.net/foxtailmillet.php, accessed on March 31, 2011) was used for alignment with the switchgrass mapped marker sequences. The parameters for BlastN program were as follows: Expected (E) threshold = 10, comparison matrix = Blosum62, alignments to show = 100, allow gaps = yes, filter = yes. The output was parsed manually to identify those significant hits with the lowest e-value and the position of each query sequence. Only the final framework markers were evaluated, and all others were omitted.

## RESULTS

### Determination of nonredundant PCR markers and polymorphism screening

**Figure 1:**
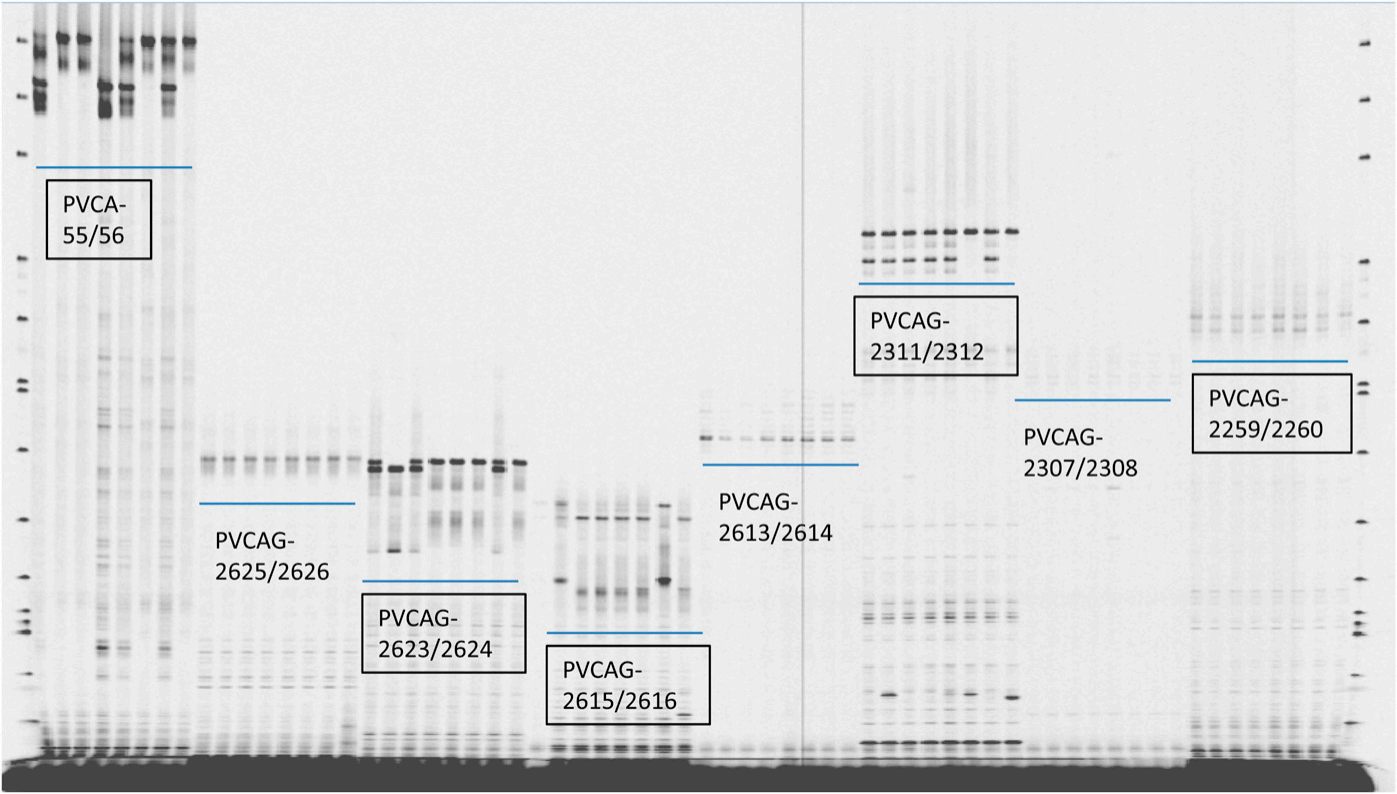
A gel image of screening SSR primer pairs for polymorphism and reliability on a panel of NL94 (first lane from left side per panel) and seven selfed progeny. Polymorphic and segregated markers are indicated in boxes. The first and last lanes are DNA ladder 50–350 size standards (LI-COR Biosciences, Lincoln, NE).

Of the 2288 switchgrass gSSR and EST SSR (eSSR) markers from different sources (Tobias *et al.* 2006, 2008; Okada *et al.* 2010; Wang *et al.* 2011), 19 were determined to be redundant PPs (see Table S2), in addition to the 4 redundant PPs reported previously (Wang *et al.* 2011). The resultant 2265 nonredundant SSR markers from switchgrass were screened for polymorphisms. Polymorphic markers were preliminarily identified if they showed segregation in the small panel of eight genotypes (Figure 1). Of 1105 switchgrass gSSRs, 377 were determined to be polymorphic. After amplifying them on a panel of 66 individuals, 7 gave monomorphic amplifications (no segregation) and 58 produced unclear amplifications, resulting in difficulties in band scoring and subsequently discarded. The remaining 312 (28.2%) were used for the linkage map construction. Of the 1160 switchgrass eSSRs, 210 showed polymorphisms in the small screening panel. Later, 48 were further discarded due to their unclear amplifications, and the remaining 162 (14%) eSSRs were used for genotyping the entire population.

To explore which marker types give more information for the linkage map construction, the relationship between polymorphism, repeat, and motif type were further analyzed. Of the 2265 nonredundant SSR markers, 1022 were of dinucleotide type, 924 were trinucleotide, 244 were compound, and the remaining 75 were tetranucleotide, pentanucleotide, hexanucleotide, or unknown repeat type. Their polymorphic rates were as high as 25.8% (63/244) for the compounds, followed by 25.1% (257/1022) for dinucleotide repeats, 16.7% (155/924) for trinucleotide, and 9.3% (7/75) belonging to other repeat types.

**Figure 2:**
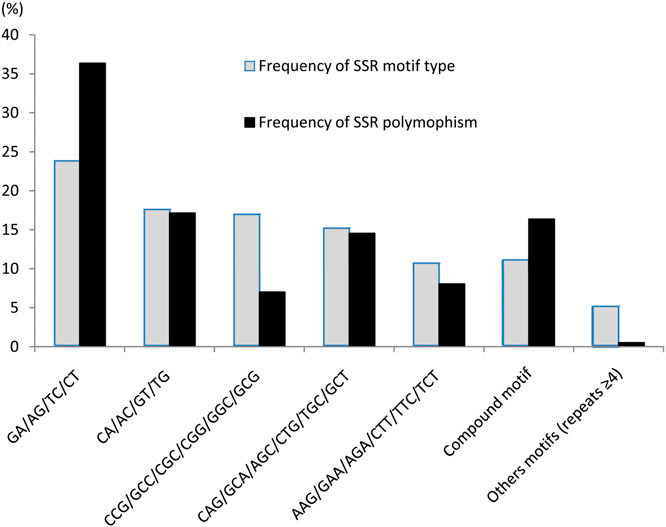
Distribution frequency and polymorphism of switchgrass SSRs based on motif types.

Among all switchgrass SSRs tested, 2221 had known motif types. Of them, GA/AG/TC/CT occurred at the highest frequency of 23.9% (531/2221), followed by CA/AC/GT/TG with 17.5% (388/2221), CCG/GCC/CGC/CGG/GGC/GCG with 16.9% [376/2221, most of them from ESTs developed by Tobias *et al.* (2008)], CAG/GCA/AGC/CTG/TGC/GCT with 15.1% (336/2221), and AAG/GAA/AGA/CTT/TTC/TCT with 10.5% (233/2221). The frequencies of motif type were almost consistent with marker polymorphisms. GA/TC was the most abundant motif type with the highest polymorphic frequency of 36.4%, followed by the motif CA/TG with 17.1% (Figure 2).

Of the 354 sorghum SSRs, 39 (11.0%) amplified clear and scorable bands in switchgrass, indicating their transferability across the species. In the small screening panel, 6 sorghum SSRs showed polymorphisms but only 1 produced clear and heritable bands and was used for the map construction. The 189 nonredundant foxtail millet SSRs were screened, and 102 (54.0%) showed expected bands and were scorable. Of them, 8 markers were validated to be effective for genotyping the entire mapping population. Together, 483 unambiguous polymorphic SSR markers, including 312 gSSR, 162 eSSR, 1 sorghum SSR, and 8 foxtail millet SSR markers, were identified to have effective polymorphisms in the population. The average number of segregated alleles per polymorphic PP was 2.03, with a range of 1 to 6 (Table 1).

**Table 1:** Amplification, polymorphism, and mean number of segregated alleles in NL94 switchgrass

Marker Types *^a^*	Marker Tested	Polymorphic SSRs	Range of Alleles	Total Amplicons from Polymorphic Primer Pairs	Total Segregated Alleles	Mean Number of Segregated Alleles in NL94	Marker Sources
SWG gSSR	1105	312	1–5	663	632	2.02	Okada *et al.* (2010); Wang *et al.* (2011)
SWG eSSR	1160	162	1–6	360	341	2.09	Tobias *et al.* (2006, 2008); Okada *et al.* (2010)
Sorghum SSR	39	1	4	4	2	2	Wu and Huang (2006)
Millet SSR	189	8	1–4	21	16	2	Jia *et al.* (2009); Dr. A. Doust (unpublished data)
Total	2493	483		1048	991	2.03 (Ave)	

a SWG, switchgrass (*Panicum virgatum*); sorghum, *Sorghum bicolor*; Millet, foxtail millet (*Setaria italica*).

### Inheritance of markers

**Figure 3:**
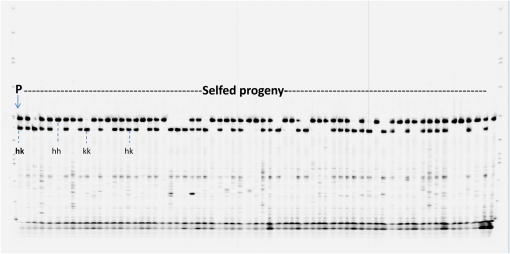
A gel image of genotyping an SSR marker PVGA-1963/1964 in the parent NL 94 (P) and 65 selfed progeny. Individual genotypes were scored as homozygous (hh or kk) or heterozygous (hk). The first and last lanes are DNA ladder 50–350 size standards (LI-COR Biosciences, Lincoln, NE).

The typical segregation of SSR markers was scored as “hh,” “hk,” or “kk” in the mapping population (Figure 3), and only three loci produced from sww-2097, sww-1678, and PVAAG-3053/3054 were scored as dominant. Of 503 codominant polymorphic loci, 81.3% (408/503) had a goodness-of-fit of 1:2:1 segregation ratio in the χ^2^ test (*P* = 0.05); the remaining 94 (18.7%) loci demonstrated distorted segregation, *i.e.* deviating from the Mendelian ratio (Table 2). Of the 3 dominant loci, 2 (sww-1678 and PVAAG-3053/3054) showed a 3:1 ratio and 1 (sww-2097 with presence:absence ratio = 80:58) deviated from 3:1.

**Table 2:** Segregation of 506 polymorphic loci in the switchgrass selfed mapping population by chi-square test

	Polymorphic Loci	Codominant		Dominant	
Marker Types		1:2:1	1:34:1	3:1	35:1
SWG gSSR	321	269 (51)*^a^*	0	1	0
SWG eSSR	176	132 (42)	0	1 (1)	0
Sorghum SSR	1	1	0	0	0
Millet SSR	8	7 (1)	0	0	0
Total	506	409 (94)	0	3 (1)	0

a Segregation-distorted loci indicated in parenthesis.

### Linkage map construction

**Figure 4:**
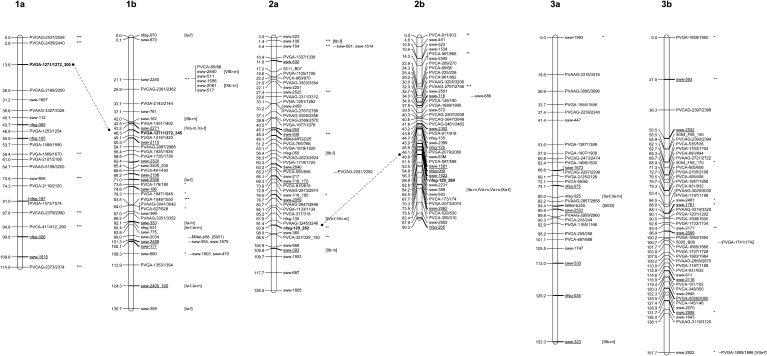

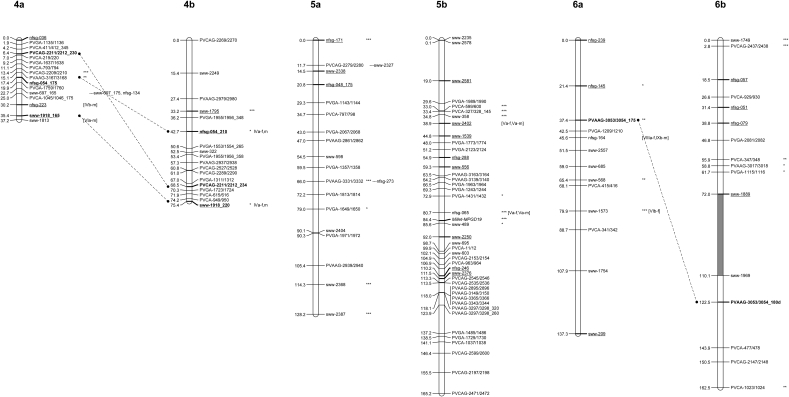

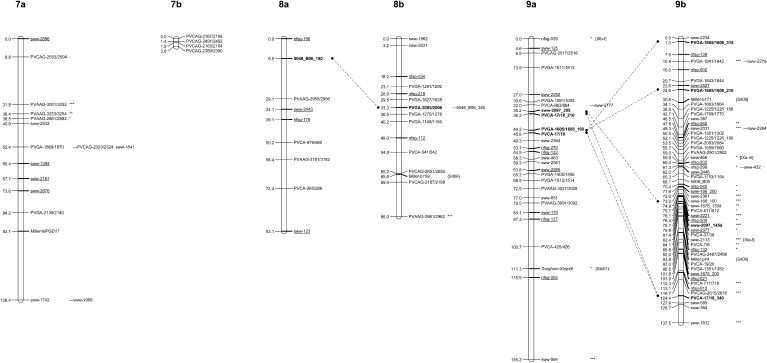
A linkage map derived from 139 self-fertilized progeny of tetraploid switchgrass NL 94. Map distances in Kosambi map units (cM) of each linkage group (LG) are shown on the left, and marker names are shown on the right. The gray segment in LG 6b indicates linkage identified with maximum linkage function at a LOD value of 2.0. The accessory loci, which are nearly equivalent to the mapped loci based on the maximum linkage function in JoinMap 4 (Van Ooijen 2006), are listed next to mapped loci. The linkage groups were grouped into homeologous groups based on multi-allele markers (in bold) and connected by dashed lines. The Arabic numeral designation of each homeologous groups (1–9) follows the previously published linkage map (Okada *et al.* 2010). The bridge markers with good collinear relationships between two switchgrass maps are underlined, and those commonly used markers but cross-linked with other LGs are denoted behind the loci with square brackets and their corresponding LGs inside. Note some markers are labeled with both symbols. The markers from sorghum *(Sorghum bicolor)* and foxtail millet *(Setaria italic)* are indicated in italics and species names are added to distinguish them from switchgrass. The dominant markers are indicated with the letter “d” appended to the marker name. The common markers shared by this map and previous sorghum (abbreviated as “sbi”) or foxtail millet (abbreviated as “Sit”) maps are labeled behind the loci with curly brackets and their corresponding LGs inside. Segregation-distorted loci (SDL) indicate different significant levels: ^*^P < 0.01, ^**^P < 0.001, and ^***^P < 0.0001.

Under the highly stringent conditions with a minimum LOD score of 8.0 and maximum REC value of 0.35, an initial framework map was constructed with 360 loci (Table 3). Then the framework order was fixed to allow the positioning of an additional 109 loci with the minimum LOD = 3.0 and maximum REC = 0.4. [This value is the maximum detectable recombination frequency for our population size of 139, according to the calculating equation of Wu *et al.* (1992)]. A lower LOD score of 2.0 was set for joining two separated groups in LG 6b because two of the markers resided in the end of each LG (sww-1969 and sww-1889) and showed a cross link with an REC frequency of 0.38. Thus, a total of 469 loci from 453 SSR PPs were ordered and placed on the final framework map with 18 LGs, the complete set expected for a tetraploid switchgrass genome (Figure 4). In addition, 30 accessory loci were assigned to likely positions on the map (Figure 4). Only 7 (1.4%) of 506 loci were not grouped or placed on the final map. Excellent correlation between the initial and final maps was observed by comparing their orders (r = 0.9873, *P* < 0.01; see Figure S2).

**Table 3:** Loci composition and recombination distance of linkage groups

Linkage Groups	Loci on Initial Framework Map*^a^*	Total Loci on Final Framework Map*^b^*	Accessory	Total Length	Average Distance (cM)	Number of Gaps >15 cM	Largest Gap per LG (cM)
1a	17	23	0	114.9	5.0	0	12.9
1b	30	38	11	135.7	3.6	1	21
2a	40	47	3	126.5	2.7	0	8.8
2b	36	42	1	90.3	2.2	0	8.9
3a	24	28	0	152.3	5.4	3	23.1
3b	35	46	2	157.7	3.4	3	21
4a	11	16	2	37.2	2.3	0	5.2
4b	12	18	0	75.4	4.1	1	15.4
5a	12	18	2	128.2	7.1	1	15.1
5b	36	43	0	165.2	3.8	1	18.9
6a	9	13	0	137.3	10.6	4	29.4
6b	10	16	0	162.5	10.2	3	38.1
7a	10	13	3	126.4	9.7	2	33.3
7b	4	4	0	3.8	1.0	0	1.9
8a	7	9	0	93.1	10.3	2	20.7
8b	13	15	1	86	5.7	1	16.6
9a	22	28	2	155.2	5.5	1	39.7*^c^*
9b	32	52	3	137.5	2.6	0	9.6
Total	360	469	30	2085.2		23	339.6
Average	20.0	26.1	1.7	115.8	4.2	1.3	

a Calculated parameters with maximum recombination ratio = 0.35 and minimum LOD = 8.0.

b Calculated parameters with maximum recombination ratio = 0.40 and minimum LOD = 3.0.

c Largest gap in whole genome.

Including accessory loci, the percentage of polymorphic markers mapped was 98.6% (499/506). The number of loci per LG varied from 4 (LG 7b) to 52 (LG 9b). The total length of the map was 2085.2 cM, and the average distance between two adjacent markers was 4.2 cM (Table 3 and Figure 4). The length of the LGs varied from 3.8 (LG 7b) to 162.5 cM (LG 6b), with an average of 115.8 cM. The marker loci were not evenly distributed across LGs, and consequently, some LGs (LG 2a, 2b, 3b, 5b, and 9b) were denser with the clustering of markers than others. Twenty-three gaps with each ≥ 15.0 cM, a distance suitable for QTL analysis and marker-assisted application (Beckmann and Soller 1983), remained and collectively spanned 339.6 cM (Table 3).

There were 120 common markers shared by this and the previous maps of Okada *et al.* (2010). Except for three local rearrangements on LG 2b and four on 9a, good collinearity of marker orders along 18 LGs was observed through 102 bridge SSR markers (Figure S3). The LGs, therefore, were named according to the previous maps (Okada *et al.* 2010) for consistency (Figure 4). The maximum number of bridge markers within each pair of corresponding LGs ranged from 3 to 16 (see Table S3). The designations of subgenome (“a” or “b”) were assigned to each LG based on the identification of bridge markers with previous maps (Okada *et al.* 2010). Among the remaining 18 common markers, 15 (83.3%) resided on their corresponding homeologous LGs based on the reference map information (Okada *et al.* 2010) (Table S3), and the other 3 were distributed in nonhomeologous LGs (Figure 4). One short LG was formed with 4 marker loci, of which none were mapped in the Okada *et al.* (2010) map. Blast analysis indicated 2 of the 4 mapped markers (*i.e.* PVCAG-2491/2492 and PVCAG-2163/2164) had hits on the bottom of sorghum chromosome 6 with the E-values of 2.2e-03 and 2.8e-05, respectively (Figure S3). Thus, it was named “LG 7b” as the sorghum chromosome 6 corresponds to switchgrass LG 7, and LG 7a was identified based on the bridge markers with the previous maps (Okada *et al.* 2010).

A polymorphic sorghum SSR (Xtxp-46), which resided on the end of sorghum LG Sbi01 (Wu and Huang 2006), was mapped on LG 9a (Figure 4). Except for a foxtail millet SSR (p58) which resided on an accessory locus of LG 1b, the other seven polymorphic foxtail millet SSRs were distributed on six different LGs, *i.e.* MPGD25 on LG 2a, b255 on LG 3a, MPGD19 on LG 5b, MPGD17 on LG 7a, b159 on LG 8b, and both b171 and p44 on LG 9b (Figure 4). A comparison of shared markers between this study and a published foxtail millet map (Jia *et al.* 2009) indicated that, except for one mismatch where a marker (Millet-b159) was expected on LG 6a (or LG 6b) but was actually mapped on LG 8b, the other seven markers showed consistent correspondence of LGs between switchgrass and foxtail millet (Figure 4).

A total of 20 multi-allele PPs were used to determine the homeologous LGs. The majority of these PPs (18 of 20) amplified one locus on each subgenome, and the remaining 2 PPs produced three different loci each. Six homeologous LG pairs (LG 1a to 1b, 2a to 2b, 4a to 4b, 6a to 6b, 8a to 8b, and 9a to 9b) were identified based on 12 shared PPs (Figure 4). The result was consistent with the LG naming system described by Okada *et al.* (2010). The other three homeologous LGs were identified based on bridge markers with the previously published maps (Okada *et al.* 2010).

### Distribution for segregation-distorted loci and the ratio of linkage phase

Among 94 segregation-distorted loci (SDL), 84.0% (79/94) were placed on the final framework map, 13.8% (13/94) belonged to accessory loci, and only 2.1% (2/94) were unmapped. Except for LG 7b and 8a, the other 16 LGs possessed SDL ranging from 3.6% (1/28) in LG 3a to 38.5% (20/52) in LG 9b (Figure 4). Clustering of SDL with greater than 4 loci was observed only in the middle part of two LGs, *i.e.* 5b and 9b. Remarkably, 10 consecutive SDL were found in LG 9b (Figure 4). The remaining SDL were randomly scattered over the LGs.

The ratio of coupling to repulsion linkage phase is expected as 1:1 for allopolyploids, and 1:0.25 in autotetraploids (Wu *et al.* 1992). Among the 469 linked loci on this framework map, a ratio of 241:228 coupling to repulsion phase linkage were detected, which conformed to a 1:1 ratio (X^2^ = 0.36 < X^2^_(1, 0.05)_ = 5.99), completely confirming the disomic inheritance reported by Liu and Wu (2011) and being highly congruent with the results of Okada *et al.* (2010).

### Mapping SSR sequences to foxtail millet genome

Of the plant species with the genome sequenced, foxtail millet is currently the closest relative of switchgrass, as both species belong to the *Paniceae* tribe and share a common ancestor 13 (±3) million years ago (Doust *et al.* 2009; Brutnell *et al.* 2010; Li and Brutnell 2011). The blast analysis indicated that of the 453 tested markers, 389 had hits in foxtail genomes. There existed a one-to-one relationship between nine switchgrass homeologous groups and nine foxtail millet chromosomes (Table 4). A total of 319 of 389 markers from the switchgrass LGs matched nine corresponding foxtail millet chromosomes (Table 4 and Table S4). Two large homeologous groups, 2 and 9 (including 71 and 62 markers, respectively), were anchored in foxtail millet chromosomes II and IX. In contrast, only 8 markers were on foxtail millet chromosome VII. The mismatched markers appeared to scatter at random on foxtail millet chromosomes.

**Table 4:** Positioning of switchgrass mapped loci in the genome of foxtail millet (*Setaria italica*)

Switchgrass Homology Group	Foxtail Millet Chromosome*^a^*										
	I	II	III	IV	V	VI	VII	VIII	IX	Total	%
1	40	3		3		1		1	2	50	80.0
2	4	71	1	1	1				2	80	88.7
3	5	3	53	1		1		1	3	67	79.1
4		3	1	18	2	2	1		1	28	64.3
5	2		1	2	37	2		1		45	82.2
6	1	1	2		1	16		1		22	72.7
7			1			1	8		1	11	72.7
8		1	1	1	1	1		14		19	73.7
9	1		2	1				1	62	67	92.5
Total	53	82	62	27	42	24	9	19	71	389 (319)*^b^*	(81.7)*^c^*

a Based on the personal communication with Dr. A. Doust (Botany Department, Oklahoma State University), the nine chromosomes of foxtail millet corresponded to the first nine assembled scaffolds, which represented 98.9% of the whole-genome sequence of foxtail millet released in Phytozome v7.0 (www.phytozome.net/foxtailmillet.php).

b Total number of loci showing one-to-one relationship between switchgrass linkage groups and foxtail millet chromosomes are indicated in parenthesis.

c Overall mean of percentage.

## DISCUSSION

### The selfed progeny population for mapping the genome in switchgrass

Due to wind-facilitated cross-pollination and strong genetic self-incompatibility, switchgrass is an allogamous species and homozygous inbred lines are unavailable in nature (Talbert *et al.* 1983). For this reason, full-sib populations from two heterozygous parents were developed to construct switchgrass linkage maps (Missaoui *et al.* 2005; Okada *et al.* 2010). The population of Missaoui *et al.* (2005) was composed of 85 full-sib progeny from a cross of ‘Alamo’ genotype AP13 (seed parent) and ‘Summer’ VS 16, whereas the population of Okada *et al.* (2010) consisted of 238 full-sib plants derived from crossing one genotype (seed parent) of “Kanlow” with a selection of “Alamo.” Because male and female meioses in the full-sib populations were distinct and independent processes, two separate parental maps were constructed, one map for the male parent and another for the female parent (Okada *et al.* 2010). Recently we identified a self-compatible lowland switchgrass genotype NL94. A first (S1) generation inbred population from selfing NL94 was developed with the assistance of marker-based identification (Liu and Wu 2011). This S1 population is similar to an F2 population derived from selfing a F1 hybrid of a cross between two different inbred lines; therefore, only one map was constructed instead of two separate (male and female) maps. In other cross-pollinated species, such as loblolly pine (Remington *et al.* 1999), sugarcane (Hoarau *et al.* 2001; Andru *et al.* 2011), and grapes (Blasi *et al.* 2011), S1 progeny have also been used for constructing linkage maps using SSR, amplified fragment length polymorphism (AFLP), resistance gene analog (RGA), and target region amplification polymorphism (TRAP) markers.

Population size is the other important issue in constructing a linkage map, because it is highly associated with the accuracy of detecting recombination events. However, the size of the mapping population is often limited by the genotyping and phenotyping costs. The population size used in this study was 139 S1 progeny resulting in 278 gametes that were used in the calculation of the recombination frequencies. Consequently, its mapping accuracy should be much higher than that used in the reference maps, as each map was constructed using 238 gametes (238 full-sib F1 individuals, Okada *et al.* 2010).

### SSR marker polymorphisms

SSRs used as a DNA marker system are advantageous over many other marker systems and have been widely utilized in many plant genomic studies (Morgante and Olivieri 1993). In this study, we exhaustively screened all available switchgrass SSR markers from various sources (Tobias *et al.* 2006, 2008; Okada *et al.* 2010; Wang *et al.* 2011) and found the polymorphic ratio of gSSRs is nearly 2-fold higher than that of eSSRs (*i.e.* 28.2% *vs.* 14.0%). This result is consistent with previous studies revealing higher polymorphism levels in gSSR than in eSSR markers (La Rota *et al.* 2005; Saha *et al.* 2005). This is because gSSRs mostly reside in nongenic regions (Varshney *et al.* 2005), so more variations can be tolerated than eSSRs. The majority of gSSRs used here were inherited in a codominant manner, and in most cases, they are chromosome-specific because only a single locus is amplified from one of the two homologous chromosomes. This unique marker-chromosome relationship is a very useful feature in a polyploidy genome. Similar phenomena were observed in wheat gSSRs (Röder *et al.* 1998a). Although previous studies indicated eSSRs are prone to accumulate in gene-rich regions and affect the coverage of linkage maps (Pinto *et al.* 2006), in this study, mapped eSSRs were interspersed along the whole linkage map, mostly between the gSSRs, and no obvious clusters were observed. It is further noteworthy that 80.6% of SSRs (2010 of 2493 tested markers) amplified monomorphic bands in the parent NL94 of this mapping population, indicating the parent has a high level of homozygosity. Higher polymorphisms of the same gSSR and eSSRs markers were reported between switchgrass varieties (Wang *et al.* 2011; Tobias *et al.* 2008). We observed a higher frequency of polymorphism in dinucleotide microsatellites (GA- and CA-motifs) than in trinucleotide microsatellites (CCG-, CAG-, and AAG-). This is in agreement with the previous results obtained in switchgrass (Wang *et al.* 2011) and other grasses, such as tall fescue (*Festuca arundinacea*) (Hirata *et al.* 2006; Saha *et al.* 2006), timothy (*Phleum pretense*) (Cai *et al.* 2003), perennial ryegrass (*Lolium perenne*) (Jones *et al.* 2001), and zoysiagrass (*Zoysia* spp.) (Cai *et al.* 2005). Trinucleotide motifs could stably reside in the target regions and suppress frameshift mutations and variations (Varshney *et al.* 2005), and dimeric repeats have been found in the untranslated regions of many species (Morgante *et al.* 2002; Jung *et al.* 2005). On the basis of the higher occurrence and polymorphism, dinucleotide SSRs could be preferentially selected for linkage map development in the future.

We observed that sorghum and foxtail millet gSSR markers had a marker transferability of 11% and 54%, respectively, which mirrored the respective divergence distance of the two species from switchgrass. Because the whole-genome sequence (although not 100%) of foxtail millet is publicly available now and has a higher transferability to switchgrass than sorghum, it provided a valuable resource to develop new molecular markers for constructing LGs of switchgrass, a plant with limited sequence information.

### Genetic linkage map

Estimates of the genome length and map distance between markers are important for the characterization of gene effects, integration of genetic and physical maps, and evaluation of map coverage. Several factors can affect the accuracy of LG construction. Among them, genotyping errors are a common factor that inflates the map length, especially when multiple bands (alleles) of the markers are scored in a polyploidy species (Pompanon *et al.* 2005). Errors can be divided into two distinct types: type I is allelic dropout, in which one allele of a heterozygote randomly fails in PCR amplification, and type II is false alleles, in which the true allele is mistakenly genotyped due to low quality of DNA, resulting in PCR or electrophoresis artifacts or scoring errors in reading and recording data (Broquet and Petit 2004). To ensure the quality of the linkage map, a number of measures were taken as follows. First, high-quality DNA samples were extracted using a stringent CTAB method (Doyle and Doyle 1990), and each sample was measured for A260/280 value to be ∼1.8 and analyzed in agarose gel electrophoresis to show only one bright band in a high molecular weight area. Second, the scoring system of codominant markers was utilized rather than the single dose dominant markers previously used by Okada *et al.* (2010) and Missaoui *et al.* (2005). We found most polymorphic markers showed mainly two reproducible alleles in parent NL94. This result is similar with previous studies where the mean number of amplicons per individual was 2.18 (Tobias *et al.* 2008). The segregation of all markers in progeny was scored as only one pattern (<hkxhk>), instead of five types (<abxcd>, <efxeg>, <lmxll>, <nnxnp>, and <hkxhk>) ranging from two to four alleles in a full-sib population. The fewer alleles recorded, the fewer chances that an allele dropout could occur, thus increasing the accuracy of genotyping. The codominant markers used here provided more information for linkage analysis than do dominant ones (Wu *et al.* 2002). Third, all marker data were scored independently by two people to reduce errors. Those markers with spurious, unstable, or redundant bands were either repeatedly tested or excluded (accounting for about 10% of total data) from the final map calculations. Four, we initially started linkage calculations using the markers with high fidelity to construct a framework map with LOD to 8.0. Those markers were fixed to allow more markers to be added on the linkage map by reducing the LOD threshold to 3.0 (except for 2.0 to link two segments of LG 6b). This strategy has been successfully used by previous studies (Wu and Huang 2006; Okada *et al.* 2010) and guaranteed both the accuracy and high coverage of the final full map.

A high collinearity between our map and the previously published switchgrass maps of crossed populations (Okada *et al.* 2010) was observed, excepting the following small discrepancies: seven inversions in marker order between our map and the reference maps (Figure S3). Except for 1 mismatched foxtail millet marker, the other 7 markers from foxtail millet (Jia *et al.* 2009) and 1 marker from sorghum (Wu and Huang 2006) matched their corresponding foxtail millet and sorghum linkage maps, respectively (Figure 4). The local rearrangement in some LGs could be real due to their universality in plant genomes (Paterson *et al.* 1996) or to genotyping errors in either of the populations. After all, errors can never be completely avoided in linkage mapping (Johnson and Haydon 2007). When a genetic map is constructed, it is assumed that marker crossovers during meiosis occur at random intervals along chromosomes (Lichten and Goldman 1995). In this study, although we used all available markers, only 4 markers were mapped on LG 7b. In contrast, LG 7b is one of the linkage groups with the highest marker density in a previous study (Okada *et al.* 2010). Of the 63 markers mapped on LG 7b by Okada *et al.* (2010), 57 markers were monomorphic and 6 markers produced unscorable bands in our population, indicating the very high homozygosity level of the NL 94 chromosome corresponding to LG 7b.

### Genome structure of switchgrass

The genome structure of switchgrass was described in previous studies, but no consistent conclusions were drawn (Missaoui *et al.* 2005; Okada *et al.* 2010; Saski *et al.* 2011), *i.e.* allotetraploid *vs.* autotetraploid. Allotetraploids have two diverged subgenomes and show the same inheritance as diploids. In this study, we speculate NL 94 switchgrass is an allotetraploid genotype because of the evidence described as follows. First, all 503 codominant markers had disomic inheritance, although 18.7% showed segregation distortion. Second, the ratio of coupling to repulsion linkage was 1:1, which is consistent with the disomic inheritance mode in polyploidy map construction (Wu *et al.* 1992; Qu and Hancock 2001). Third, among 476 mapped SSR markers, only 20 (4.2%) amplified more than two bands and mapped on homeologous LGs, indicating that the two subgenomes are significantly different from each other. The allotetraploid-like genome structure of the tetraploid switchgrass uncovered by this study and the two recent molecular marker investigations (Okada *et al.* 2010; Liu and Wu 2011) agree well with the published cytological observations of the bivalent pairing behavior of meiotic chromosomes (Barnett and Carver 1967; Martinez-Reyna *et al.* 2001; Young *et al.* 2010). However, because diploid wild ancestors of switchgrass are unknown and there are many examples of autotetraploids that through time have undergone “diploidization” leading to disomic inheritance (see review by Wolfe 2001), much more sequence data are needed to address this issue of allopolyploidy confidently.

In this study, we successfully identified six homeologous LGs using the 20 multi-allele markers. Attempts were made to find more multi-allele markers, but most of those multi-allele primer pairs were error-prone during band scoring or unstable for the entire population. To avoid eroding the quality of the linkage map, we were cautious when selecting these markers. Approximately 20% the eSSR markers amplified alleles in two subgenomes of switchgrass in previous maps and identified nine complete homeologous LGs (Okada *et al.* 2010). Although higher numbers of multi-allele SSRs were reported in their study, Okada *et al.* (2010) strongly believed that the subgenomes of switchgrass were distantly related. Through the bridge markers and comparative mapping between ours and published maps (Okada *et al.* 2010), nine complete homeologous LGs were identified in this study.

### Segregation distortion

Numerous examples of segregation distortion have been reported in many crop species, including barley (*Hordeum vulgare*) (Graner *et al.* 1991; Devaux *et al.* 1995), rice (*Oryza sativa*)(Causse *et al.* 1994; Xu *et al.* 1997), maize (*Zea mays*) (Wendel *et al.* 1987; Lu *et al.* 2002), and wheat (*Triticum aestivum*) (Blanco *et al.* 2004; Quarrie *et al.* 2005). In this study, segregation distortion was observed for 18.7% of the total marker loci analyzed. This is slightly higher than that of the full-sib switchgrass population with a value of up to 14% (Okada *et al.* 2010). Because a selfed population was used here, the result is consistent with previous reports that showed selfed populations had a higher tendency for segregation distortions due to inbreeding depression (Andru *et al.* 2011; Charlesworth and Willis 2009).

Two SDL clusters were identified on LG 5b and 9b in this study (Figure 4). Previous studies indicated that segregation distortion was an indication of the linkage between molecular markers and distorting factors (such as recessive lethal genes and incompatible alleles) (Lyttle 1991). If the linkage was tight, they usually had similar segregation patterns and the skewed markers would appear to be clustered (Jenczewski *et al.* 1997). These two SDL clusters were not found in other studies, suggesting they are population-specific. Similarly, in sugarcane and grape S1 maps (Hoarau *et al.* 2001; Blasi *et al.* 2011), some distorted markers were also clustered together.

### Comparative mapping

Comparative genome relationships have been established among rice, foxtail millet, sugar cane, sorghum, pearl millet, maize, and the *Triticeae* cereals (wheat, barley, rye, and oats) (Gale and Devos 1998). As an emerging biofuel crop with a relatively short breeding history, switchgrass had the only reported relationship with the sorghum genome, in which nine homeologous groups of switchgrass corresponded to 10 sorghum chromosomes (Okada *et al.* 2010). Here we utilized the newly released foxtail millet sequence and revealed that about 80% of switchgrass markers were located in the foxtail millet chromosomes. This result directly indicated the strong one-to-one relationship between the chromosomes of switchgrass and foxtail millet. The corresponding genomic relationship among switchgrass, foxtail millet, and sorghum is consistent with a previously integrated grass comparative map (Devos and Gale 1997). The high genetic similarity between switchgrass and foxtail millet is not surprising when considering their close relationship, with both belonging to the same *Paniceae* tribe and the high collinearity among grasses reported previously (Devos and Gale 1997, 2000). However, mismatched markers still existed. These might have been derived from chromosomal rearrangements occurring after the respective speciation of both switchgrass and foxtail millet. Due to its small stature, rapid life cycle, inbred pollination, and prolific seed production, foxtail millet is a desirable candidate to be used as a reference model for switchgrass genetic and functional genomics investigation.

### Genomics and breeding implications

Switchgrass has large, highly heterozygous polyploidy genomes that hinder the effort of whole-genome sequencing. Dihaploid lines were identified in switchgrass (Young *et al.* 2010) and seem to be helpful for simplifying sequence assembly but are unsuitable for entire genome sequencing due to their high sterility and instability (Young *et al.* 2010). A diploid relative of switchgrass (*i.e.* *Panicum hallii*) (2n = 2x = 18) is being sequenced at the Joint Genome Institute (JGI, http://www.jgi.doe.gov/genome-projects/); however, it has only one set of chromosomes, and therefore, it may not be effective for defining two subgenomes of switchgrass. Here we constructed a high-density genetic map derived from selfing a single genotype NL94. A total of 476 SSR markers were mapped in more than 2000 cM genome length with average marker distance of 4.2 cM. The present map provides an excellent tool for distinguishing switchgrass subgenomes. Through continuing to self switchgrass plants like NL94, inbred lines are being produced in our lab. These could be used as the starting material for whole-genome sequencing.

Based on our preliminary observations in the field, the selfed progeny of NL94 showed significant segregation for biomass and its associated traits, such as plant height, circumference of plant base, tiller number, and spring growth vigor. It would be much easier to perform a QTL analysis using a single map than two maps from a full-sib population. Our S1 mapping population should have some features of F2 populations, including the estimation of both additive and dominant effects (Carbonell *et al.* 1993).

In conclusion, this study developed a complete genetic map of 18 linkage groups in an inbred lowland switchgrass population using mostly codominant SSR markers. This map is the longest and most dense one constructed to date. Two clusters of segregation-distorted loci were found in the switchgrass genome. A one-to-one relationship between nine switchgrass homeologous groups and nine foxtail millet chromosomes was established. This linkage map should provide a new genetic framework to facilitate genomics research, quantitative trait locus (QTL) mapping, and marker-assisted selection.

## Supplementary Material

Supporting Information
