# Supplementary material for: A High-Density Simple Sequence Repeat-Based Genetic Linkage Map of Switchgrass
Source: G3 (Bethesda). 2012 Mar 1;2(3):357–70. doi: 10.1534/g3.111.001503 (PMC3291506; doi:10.1534/g3.111.001503)
Supplement: Supporting Information [file supp_2_3_357__index.html]

Supporting Information 

# A High-Density Simple Sequence Repeat-Based Genetic Linkage Map of Switchgrass

## Supporting Information for Liu *et al.*, 2012

**Files in this Data Supplement:**

- Supporting Information - Figures S1-S3, Tables S1-S4, and File S1 (PDF, 2.9 MB)
- Figure S1 - Detection of 49 bp deletion in *trnL-UAA* intron in lowland and upland switchgrass (PDF, 180 KB)
- Figure S2 - Collinerity between the initial and final framework linkage maps (PDF, 140 KB)
- Figure S3 - Collinerity between this and published linkage map (Okada *et al.* 2010) (PDF, 2.4 MB)
- Table S1 - Expected progeny phenotypes and segregation ratios for disomic and tetrasomic inheritance in a selfed population of a tetraploid switchgrass plant (PDF, 84 KB)
- Table S2 - Detection of redundancy among published primers (Tobias et al. 2006, 2008; Wang et al. 2011) using bl2seq basic local alignment search tool (BLAST) (NCBI, Bethesda, MD) (PDF, 88 KB)
- Table S3 - Presentation of identical bridge markers in the linkage groups (LGs) of this map and a previous study (Okada et al. 2010) (PDF, 79 KB)
- Table S4 - A list of switchgrass markers and their corresponding nine chromosomes of foxtail millet by BlastN analysis in Phytozome v7.0 (PDF, 79 KB)
- File S1  - Raw Genotyping Data (.txt, 249 KB)
