# Supplementary material for: A High-Density Simple Sequence Repeat-Based Genetic Linkage Map of Switchgrass
Source: G3 (Bethesda). 2012 Mar 1;2(3):357–70. doi: 10.1534/g3.111.001503 (PMC3291506; doi:10.1534/g3.111.001503)
Supplement: Supporting Information [file supp_2.3.357_001503SI.pdf]

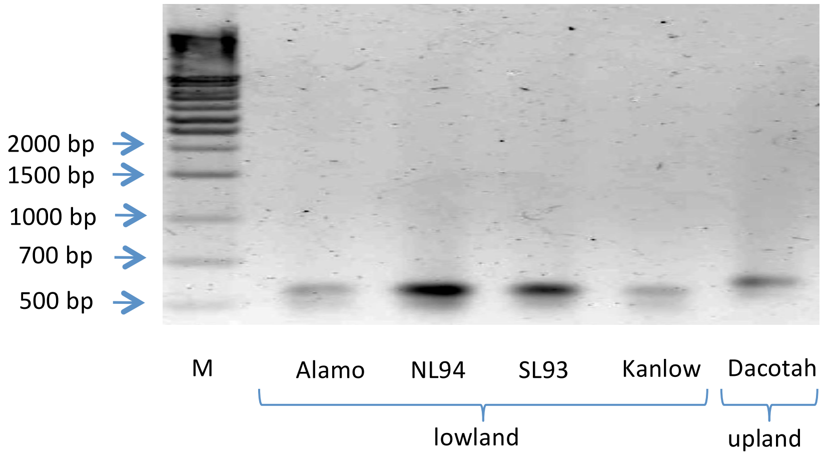

Figure S1 Detection of 49 bp deletion in *trnL-UAA* intron in lowland and upland switchgrass. Alamo and Kanlow represented the southern and northern lowland ecotypes, respectively. While Dacotah represented a typical upland ecotype. "M" indicated the molecular weight standard with 1 kb DNA Ladder (Axygen Biosciences, Union City, CA)

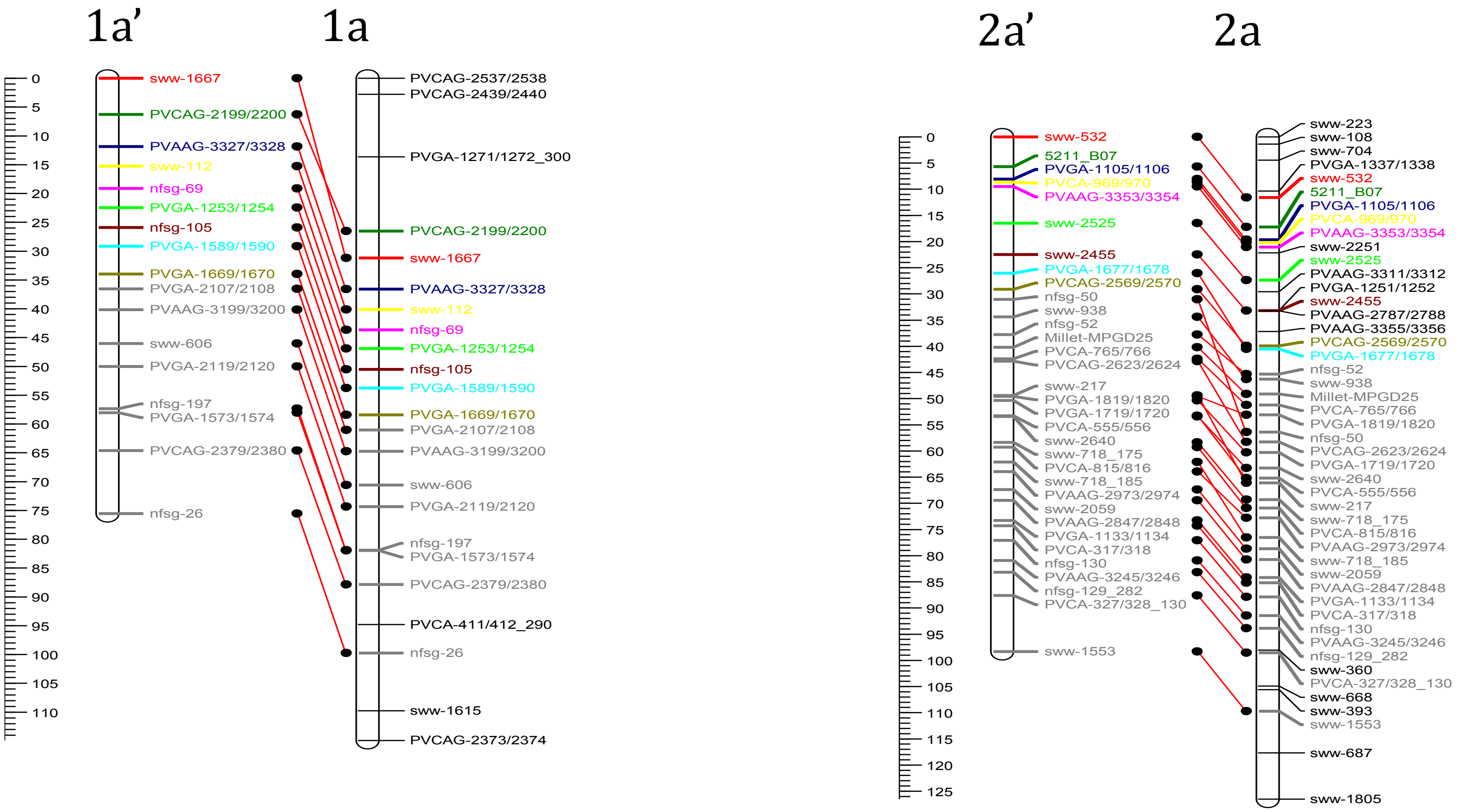

Figure S2 Collinerity between the initial and final framework linkage maps. Here only linkage groups (LGs) 1a and 2a are shown as the representatives of all LGs. The LG number of initial framework map is indicated by a quote symbol. All loci mapped with the same markers across LGs are highlighted with matching colors

Figure S3

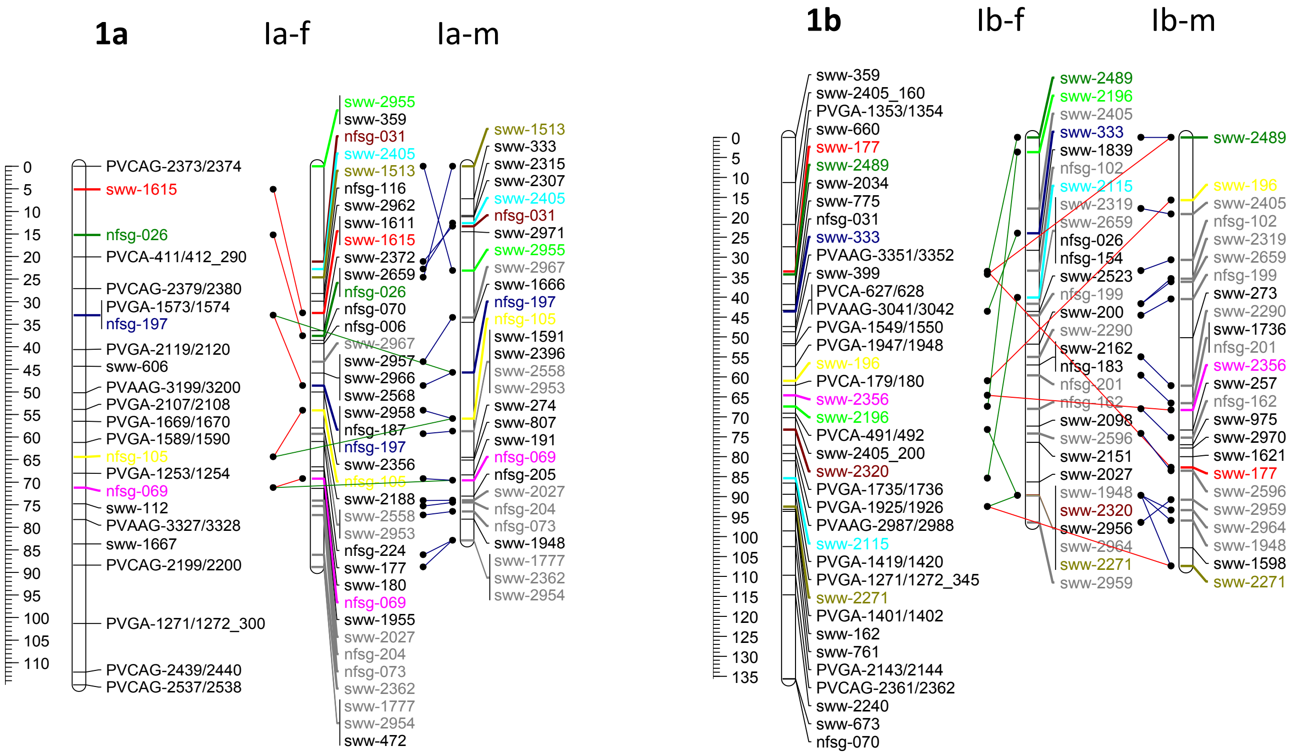

1a
Ia-f
Ia-m
PVCAG-2373/2374
sww-1615
nfsg-026
PVCA-411/412_290
PVCAG-2379/2380
PVGA-1573/1574
nfsg-197
PVGA-2119/2120
sww-606
PVAAG-3199/3200
PVGA-2107/2108
PVGA-1669/1670
PVGA-1589/1590
nfsg-105
PVGA-1253/1254
nfsg-069
sww-112
PVAAG-3327/3328
sww-1667
PVCAG-2199/2200
PVGA-1271/1272_300
PVCAG-2439/2440
PVCAG-2537/2538
sww-2955
sww-359
nfsg-031
sww-2405
sww-1513
nfsg-116
sww-2962
sww-1611
sww-1615
sww-2372
sww-2659
nfsg-026
nfsg-070
nfsg-006
sww-2967
sww-2957
sww-2966
sww-2568
sww-2958
nfsg-187
nfsg-197
sww-2356
nfsg-105
sww-2188
sww-2558
sww-2953
nfsg-224
sww-177
sww-180
nfsg-069
sww-1955
sww-2027
nfsg-204
nfsg-073
sww-2362
sww-1777
sww-2954
sww-472
sww-1513
sww-333
sww-2315
sww-2307
sww-2405
nfsg-031
sww-2971
sww-2955
sww-2967
sww-1666
nfsg-197
nfsg-105
sww-1591
sww-2396
sww-2558
sww-2953
sww-274
sww-807
sww-191
nfsg-069
nfsg-205
sww-2027
nfsg-204
nfsg-073
sww-1948
sww-1777
sww-2362
sww-2954
1b
Ib-f
Ib-m
sww-359
sww-2405_160
PVGA-1353/1354
sww-660
sww-177
sww-2489
sww-2034
sww-775
nfsg-031
sww-333
PVAAG-3351/3352
sww-399
PVCA-627/628
PVAAG-3041/3042
PVGA-1549/1550
PVGA-1947/1948
sww-196
PVCA-179/180
sww-2356
sww-2196
PVCA-491/492
sww-2405_200
sww-2320
PVGA-1735/1736
PVGA-1925/1926
PVAAG-2987/2988
sww-2115
PVGA-1419/1420
PVGA-1271/1272_345
sww-2271
PVGA-1401/1402
sww-162
sww-761
PVGA-2143/2144
PVCAG-2361/2362
sww-2240
sww-673
nfsg-070
sww-2489
sww-2196
sww-2405
sww-333
sww-1839
nfsg-102
sww-2115
sww-2319
sww-2659
nfsg-026
nfsg-154
sww-2523
nfsg-199
sww-200
sww-2290
sww-2162
nfsg-183
nfsg-201
nfsg-162
sww-2098
sww-2596
sww-2151
sww-2027
sww-1948
sww-2320
sww-2956
sww-2964
sww-2271
sww-2959
sww-2489
sww-196
sww-2405
nfsg-102
sww-2319
sww-2659
nfsg-199
sww-273
sww-2290
sww-1736
nfsg-201
sww-2356
sww-257
nfsg-162
sww-975
sww-2970
sww-1621
sww-177
sww-2596
sww-2959
sww-2964
sww-1948
sww-1598
sww-2271

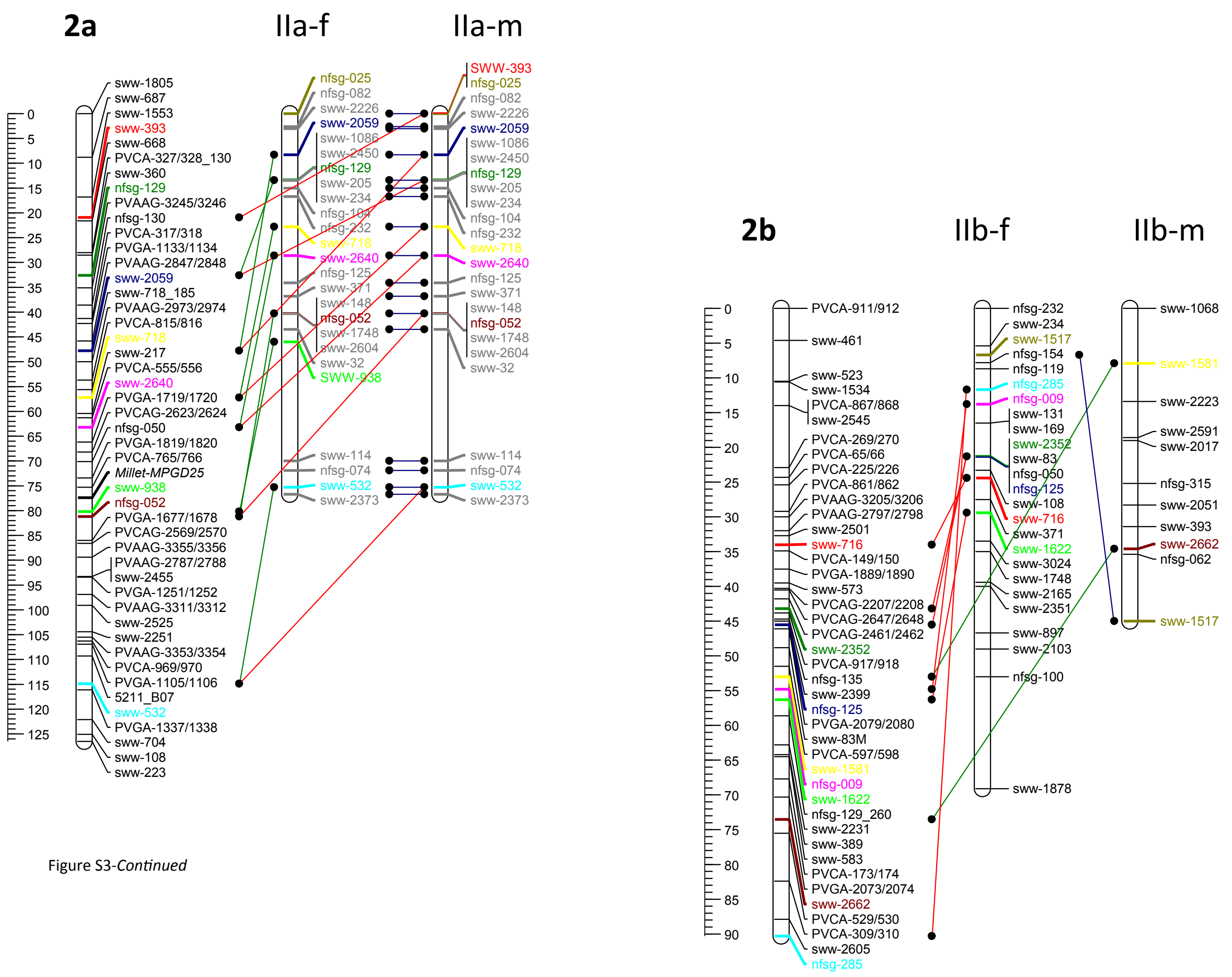

Figure S3-*Continued*

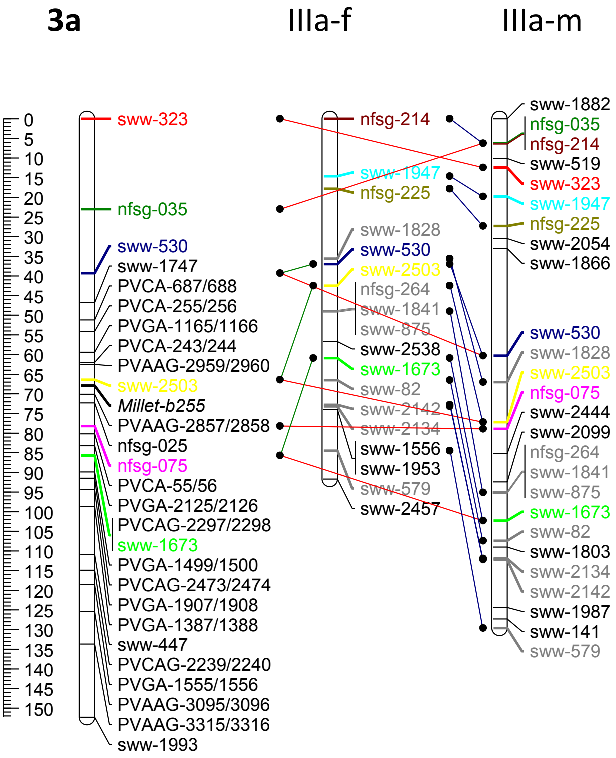

Figure S3-*Continued*

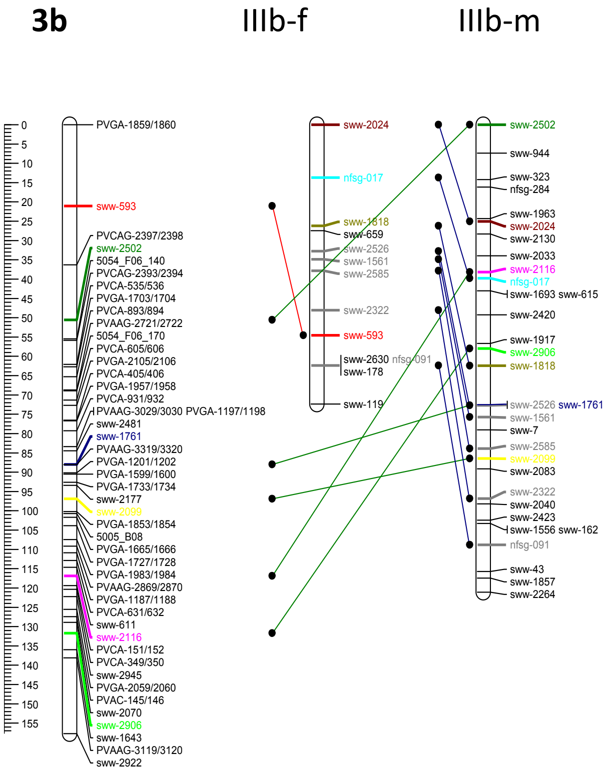

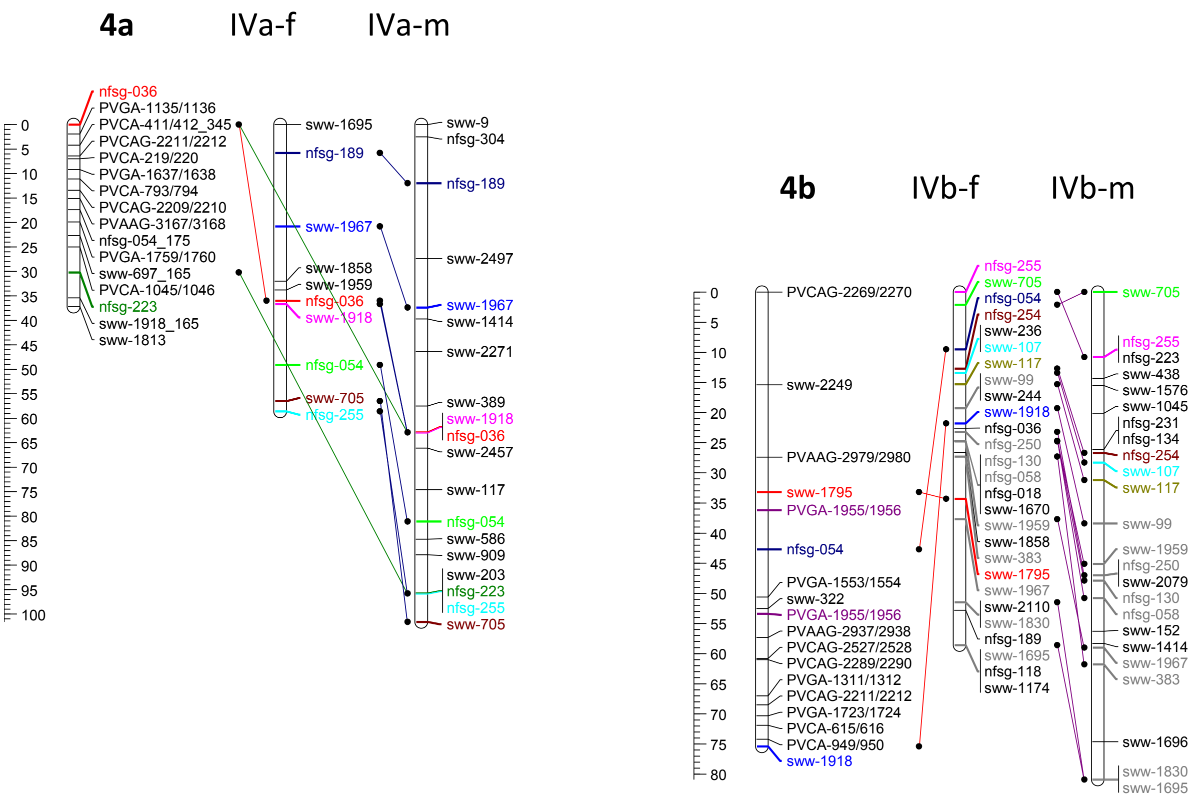

Figure S3-*Continued*

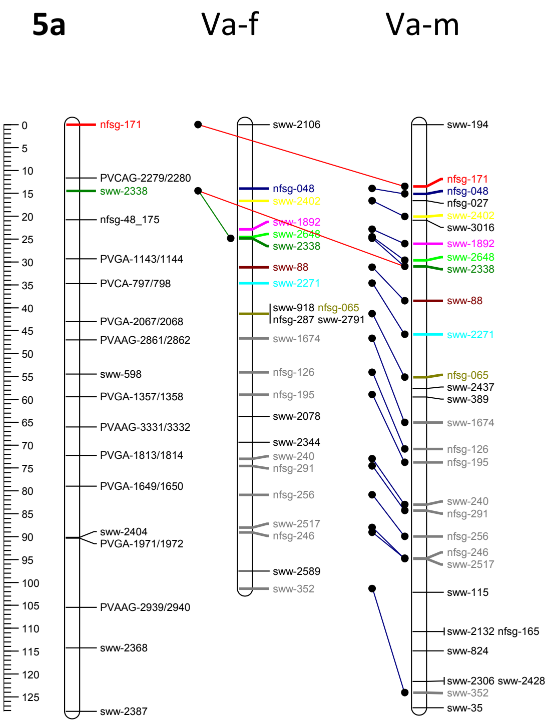

Figure S3-*Continued*

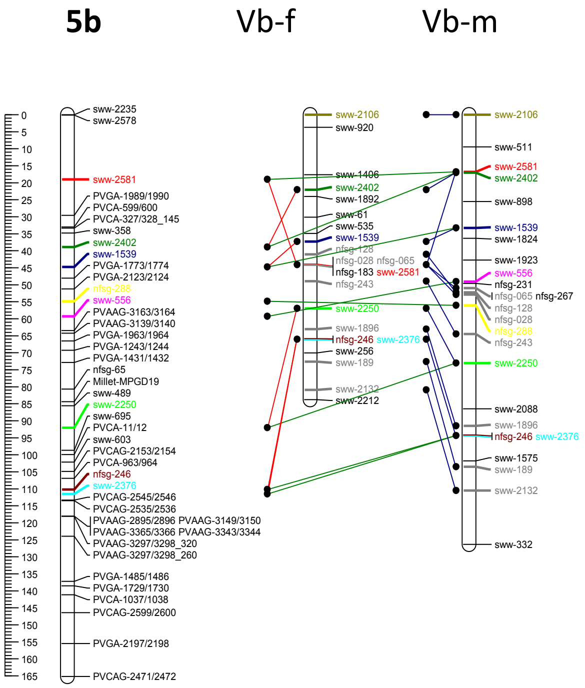

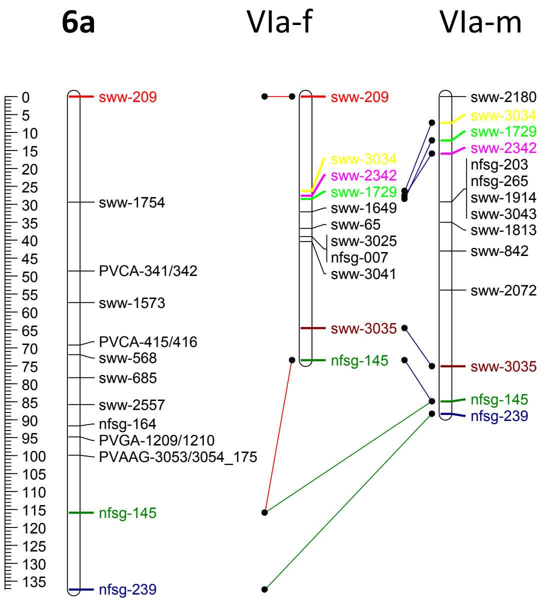

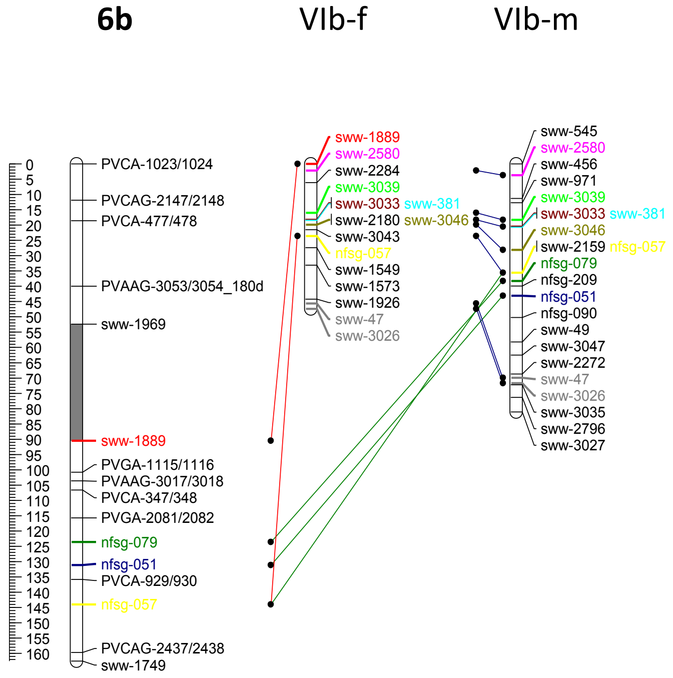

Figure S3-*Continued*

7a

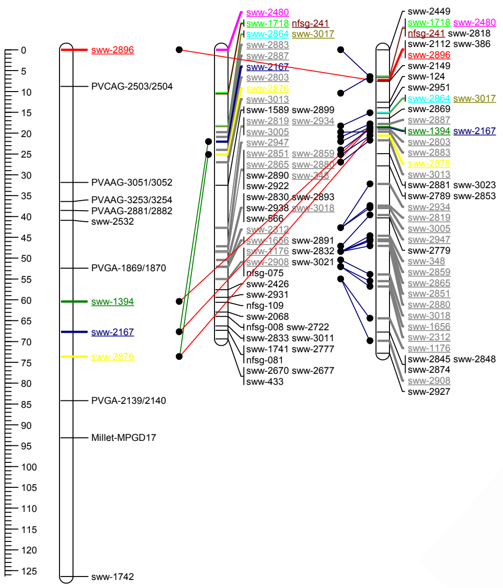

7b

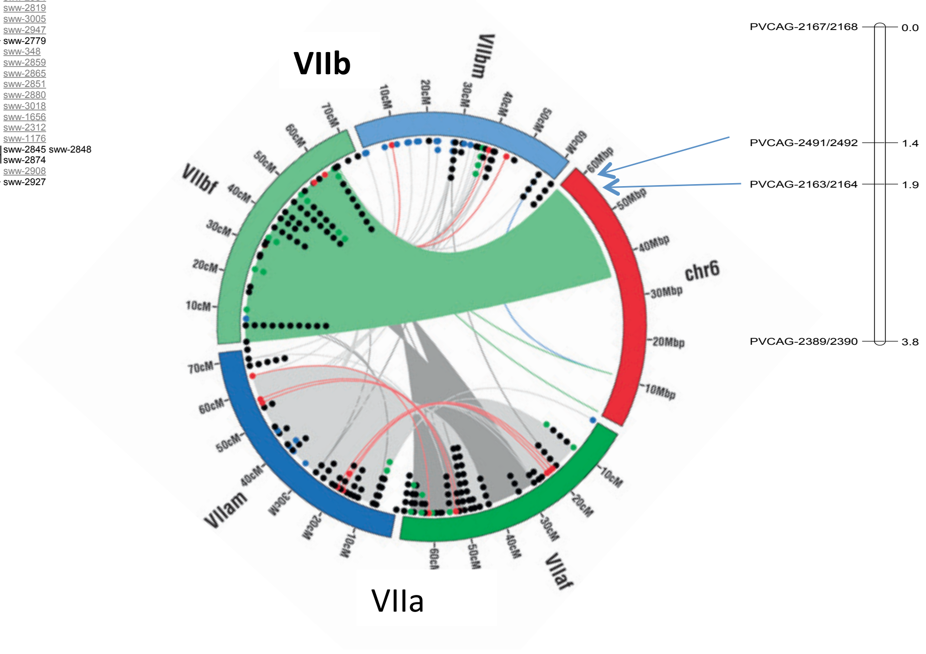

Figure S3-*Continued*

Circos image was taken from Okada *et al*. 2010 Genetics. Doi:10.1534/genetics. 110.113910

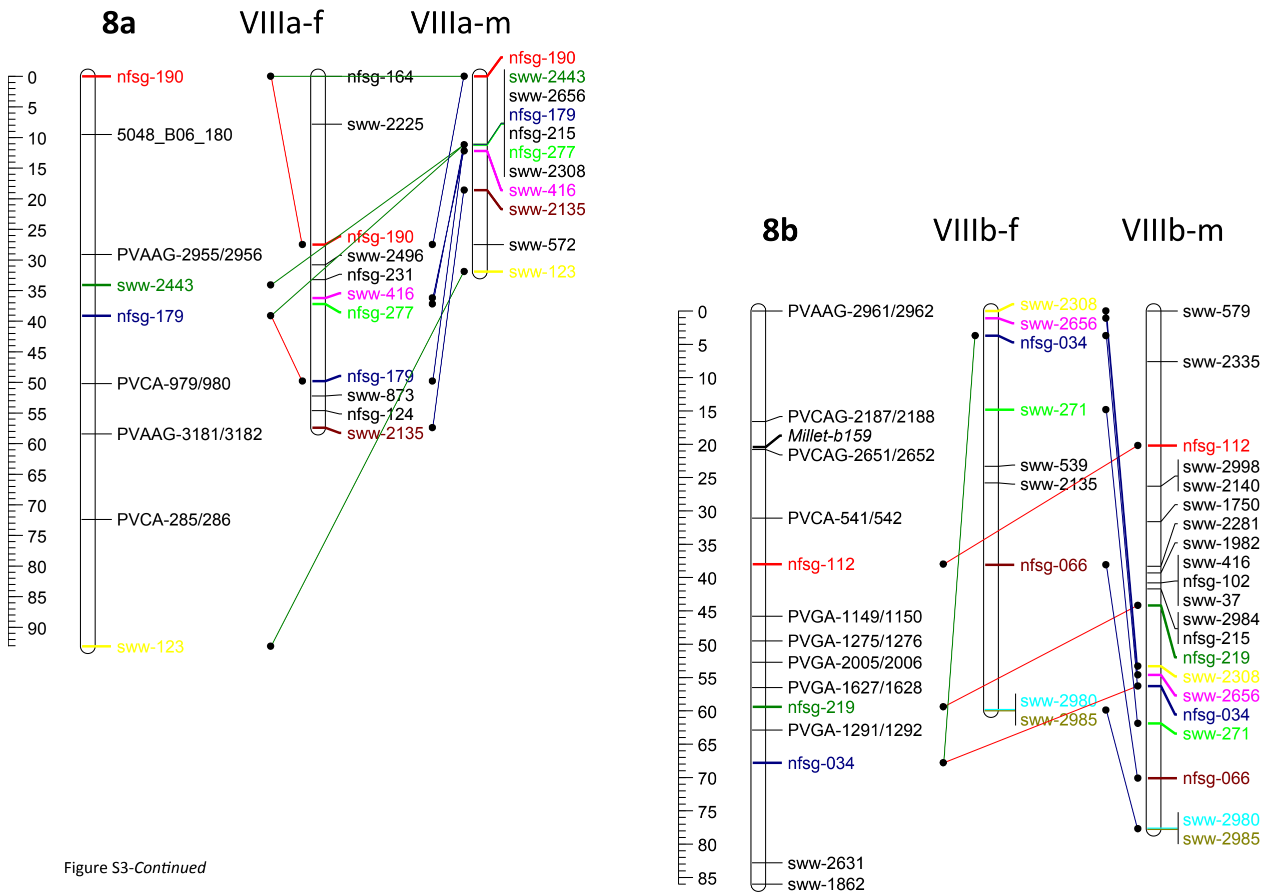

Figure S3-*Continued*

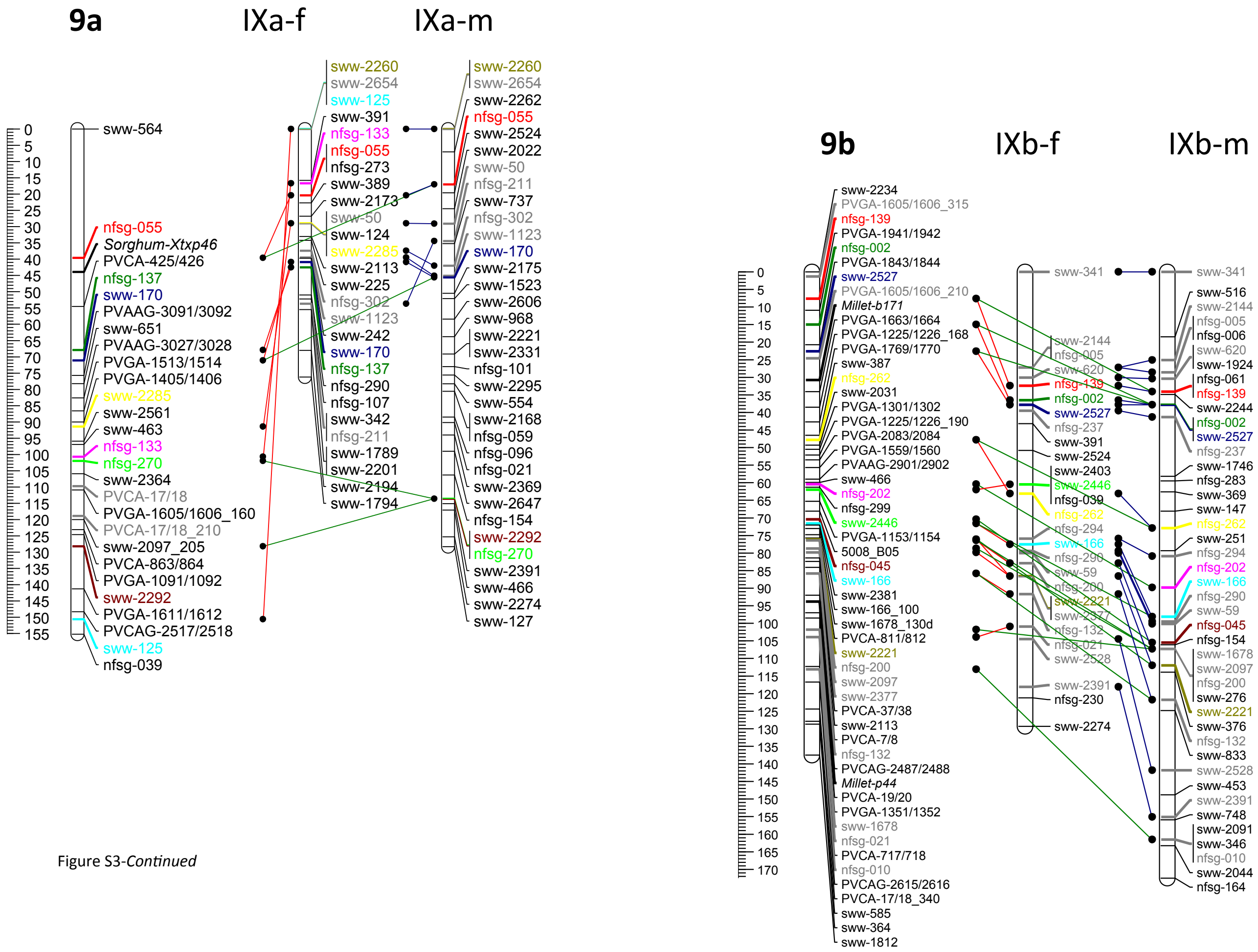

Figure S3-*Continued*

Figure S3 Collinerity between this and published linkage map (Okada *et al.* 2010). The names of linkage groups (LGs) in this study are indicated with Arabic numbers in bold, and the designation of LGs and two subgenomes ('a' and 'b') is consistent with the reference map (Okada *et al.* 2010). The letter 'f' and 'm' at the end of reference LGs (Roman number) denotes female and male parent maps, respectively. To compare the two maps, the duplicated loci amplified from same markers in reference map and the band sizes were removed. All loci mapped with the same markers across LGs are highlighted with matching colors.

File S1

Raw Genotyping Data

File S1 is available for download at http://www.g3journal.org/lookup/suppl/doi:10.1534/g3.111.001503/-/DC1.

Table S1 Expected progeny phenotypes and segregation ratios for disomic and tetrasomic inheritance in a selfed population of a tetraploid switchgrass plant

Inheritance mode	Marker scoring types	Genotypes of two bands observed [a]	Theoretical genotypes of selfed progeny [b]	Expected selfed progeny band pattern	Expected progeny ratio
Disomic	co-dominant	*AB*	*1AA, 2AB, 1BB*	a:ab:b	1:2:1
Tetrasomic		*ABBB*	*(1AABB,2ABBB),1BBBB*	ab:b	3:1
		AABB	*1AAAA,(8AAAB,18AABB,8ABBB),1BBBB*	a:ab:b	1:34:1
		AAAB	*1AAAA,(2AAAB,1AABB)*	a:ab	1:3
Disomic	dominant	*A_*	(*1AA, 2A_*), *1_ _*	presence:absence	3:1
Tetrasomic		*A_ _ _*	*(1AA_ _ ,2A_ _ _),1_ _ _ _*	presence:absence	3:1
		AA_ _	*(1AAAA,8AAA_ ,18AA_ _ ,8A_ _ _),1_ _ _ _*	presence:absence	35:1
		*AAA_*	(1AAAA,2AAA_ ,1AA_ _)	presence:absence	all presence

[a] Italicized capital letters represent genotypes.
[b] The genotypes in parenthesis produce the same gel banding phenotypes.

Table S2 Detection of redundancy among published primers (Tobias *et al.* 2006, 2008; Wang *et al.* 2011) using bl2seq basic local alignment search tool (BLAST) (NCBI, Bethesda, MD)

Marker ID of Wang et al. (2011)	Marker ID of Okada et al. (2010)	SSR type	% Read (similarity)	Read length (bp)	E value	Bit score [b]
PVGA-1225/1226	NFSG005	gSSR	100	21	9.00E-08	39.9
PVGA-1901/1902	NFSG006	gSSR	100	22	6.00E-08	41.7
PVGA-1643/1644	NFSG066	gSSR	100	21	4.00E-09	39.9
PVGA-1419/1420	NFSG102	gSSR	100	20	2.00E-10	38.1
PVGA2-1329/1330	NFSG136	gSSR	100	20	7.00E-09	38.1
	NFSG249	gSSR	100	20	5.00E-08	39.9
PVGA-1669/1670	NFSG187	gSSR	100	21	4.00E-08	39.9
PVGA-1627/1628	NFSG215	gSSR	100	21	2.00E-09	38.9
PVGA-1657/1658	NFSG243	gSSR	100	21	1.00E-08	30.7
PVGA-1867/1868	SWW2864	eSSR	100	20	2.30E-08	39.9
PVGA-2035/2036	SWW2881	eSSR	100	21	4.00E-08	39.9
PVGA-1295/1296	SWW2954	eSSR	100	21	7.00E-09	38.8
PVCAG-2245/2246	SWW3033	eSSR	100	21	2.10E-09	30.7
PVGA6-1793/1794	SWW2839	eSSR	76	16	4.00E-06	38.8
PVGA5-1697/1698	sww2845	eSSR	100	21	1.41E-09	38.8
PVCA- 985/986	SWW3043	eSSR	100	18	8.10E-07	38.1
sww1 [a]	4821_A04	eSSR	100	20	2.00E-09	38.1
sww842	4934_H04	eSSR	100	20	1.00E-09	38.1
SWW35	No reverse sequence hit in GenBank	eSSR				
NFSG028	Redundant with NFSG027	gSSR				

[a] The last four primer pairs are the comparison of primer pairs from same research group (Tobias *et al.* 2006, 2008; Okada *et al.* 2010)

[b] *Note* the bit score represents the normalized value that can be used to compare alignment scores from different searches

Table S3 Presentation of identical bridge markers in the linkage groups (LGs) of this map and a previous study (Okada *et al*. 2010)

LGs in this study	Corresponding LGs and subgenome designation of Okada *et al*. 2010	No. of bridge markers	LGs and alternate subgenome designation of Okada *et al*. 2010	No. of bridge markers
1a	Ia	5	Ib	0
1b	Ib	10	Ia	5
2a	IIa	8	IIb	2
2b	IIb	8	IIa	1
3a	IIIa	6	IIIb	1
3b	IIIb	7	IIIa	0
4a[a]	IVa	4	IVb	1
4b	IVb	3	IVa	2
5a	Va	3	Vb	0
5b	Vb	8	Va	2
6a	VIa	3	VIb	1
6b	VIb	4	Via	0
7a	VIIa	4	VIIb	0
7b	VIIb	0	VIIa	0
8a	VIIIa	4	VIIIb	0
8b	VIIIb	3	VIIIa	0
9a	IXa	8	IXb	1
9b	IXb	16	Ixa	2
Total		**102**		**15**

[a] Two bridge markers (nfsg-054 and sww-1918) were shared by both LG 4a and 4b.

Table S4 A list of switchgrass markers and their corresponding nine chromosomes of foxtail millet by BlastN analysis in Phytozome v7.0

Chr. of foxtail millet	Switchgrass markers	Total
I	nfsg-026,nfsg-105,nfsg-197,PVAAG-2987/2988,PVAAG-3327/3328,PVAAG-3351/3352,PVCA-179/180,PVCA-411/412,PVCA-627/628,PVCAG-2199/2200,PVCAG-2379/2380,PVCAG-2439/2440,PVCAG-2537/2538,PVGA-1253/1254,PVGA-1271/1272,PVGA-1353/1354,PVGA-1573/1574,PVGA-1669/1670,PVGA-1735/1736,PVGA-1925/1926,PVGA-1947/1948,PVGA-2119/2120,sww-112,sww-1615,sww-162,sww-1667,sww-177,sww-196,sww-2115,sww-2196,sww-2240,sww-2271,sww-2320,sww-2405,sww-2489,sww-333,sww-359,sww-606,sww-660,sww-673	**40**
II	PVCA-529/530,5211_B07,Millet-MPGD25,nfsg-050,nfsg-052,nfsg-125,nfsg-129,nfsg-130,nfsg-285,PVAAG-2787/2788,PVAAG-2797/2798,PVAAG-2847/2848,PVAAG-3205/3206,PVAAG-3245/3246,PVAAG-3311/3312,PVAAG-3353/3354,PVAAG-3355/3356,PVCA-173/174,PVCA-269/270,PVCA-309/310,PVCA-317/318,PVCA-327/328,PVCA-555/556,PVCA-597/598,PVCA-765/766,PVCA-815/816,PVCA-861/862,PVCA-867/868,PVCA-911/912,PVCA-917/918,PVCA-969/970,PVCAG-2207/2208,PVCAG-2461/2462,PVCAG-2569/2570,PVCAG-2623/2624,PVGA-1105/1106,PVGA-1133/1134,PVGA-1337/1338,PVGA-1677/1678,PVGA-1719/1720,PVGA-1819/1820,PVGA-2079/2080,sww-108,sww-1553,sww-1581,sww-1622,sww-1805,sww-217,sww-223,sww-2231,sww-2251,sww-2352,sww-2455,sww-2501,sww-2525,sww-2545,sww-2640,sww-2662,sww-360,sww-389,sww-393,sww-461,sww-523,sww-532,sww-573,sww-583,sww-668,sww-704,sww-716,sww-718,sww-938	**71**
III	5005_B08,5054_F06,Millet-b255,nfsg-025,nfsg-035,nfsg-075,PVAAG-2721/2722,PVAAG-2869/2870,PVAAG-2959/2960,PVAAG-3095/3096,PVAAG-3119/3120,PVAAG-3315/3316,PVAAG-3319/3320,PVCA-151/152,PVCA-405/406,PVCA-55/56,PVCA-631/632,PVCA-687/688,PVCA-893/894,PVCA-931/932,PVCAG-2239/2240,PVCAG-2297/2298,PVCAG-2393/2394,PVCAG-2473/2474,PVGA-1115/1116,PVGA-1187/1188,PVGA-1197/1198,PVGA-1201/1202,PVGA-1665/1666,PVGA-1703/1704,PVGA-1727/1728,PVGA-1957/1958,PVGA-1983/1984,PVGA-2059/2060,PVGA-2105/2106,PVGA-2125/2126,sww-1643,sww-1673,sww-1761,sww-1993,sww-2070,sww-2177,sww-2481,sww-2502,sww-2503,sww-2906,sww-2922,sww-2945,sww-323,sww-447,sww-530,sww-593,sww-611	**53**
IV	PVCA-1045/1046,nfsg-036,nfsg-054,PVAAG-3167/3168,PVCA-219/220,PVCA-615/616,PVCA-793/794,PVCA-949/950,PVCAG-2269/2270,PVCAG-2527/2528,PVGA-1311/1312,PVGA-1553/1554,PVGA-1637/1638,PVGA-1723/1724,PVGA-1759/1760,PVGA-1955/1956,sww-1795,sww-697	**18**
V	PVCA-1037/1038,Millet-MPGD19,nfsg-048,nfsg-065,nfsg-246,PVAAG-2861/2862,PVAAG-2895/2896,PVAAG-2939/2940,PVAAG-3139/3140,PVAAG-3149/3150,PVAAG-3331/3332,PVCA-11/12,PVCA-797/798,PVCAG-2153/2154,PVCAG-2197/2198,PVCAG-2279/2280,PVCAG-2471/2472,PVCAG-2535/2536,PVCAG-2545/2546,PVGA-1143/1144,PVGA-1243/1244,PVGA-1963/1964,PVGA-1989/1990,PVGA-2123/2124,sww-1539,sww-2235,sww-2338,sww-2368,sww-2376,sww-2387,sww-2402,sww-2404,sww-2578,sww-2581,sww-358,sww-598,sww-603	**37**
VI	PVCA-1023/1024,nfsg-145,nfsg-164,nfsg-239,PVAAG-3017/3018,PVAAG-3053/3054,PVCA-341/342,PVGA-1209/1210,sww-1754,sww-1889,sww-1969,sww-209,sww-2557,sww-568,sww-685	**16**
VII	Millet-MPGD17,PVCAG-2163/2164,PVCAG-2389/2390,PVCAG-2503/2504,PVGA-1869/1870,sww-1394,sww-1742,sww-2532	**8**
VIII	5048_B06,nfsg-034,nfsg-112,nfsg-219,PVAAG-2961/2962,PVAAG-3181/3182,PVCA-541/542,PVCAG-2187/2188,PVGA-1149/1150,PVGA-1275/1276,PVGA-1627/1628,sww-123,sww-1862,sww-2443	**14**
IX	5008_B05,Millet-b171,Millet-p44,nfsg-010,nfsg-039,nfsg-055,nfsg-132,nfsg-133,nfsg-200,nfsg-202,nfsg-262,nfsg-270,PVAAG-2901/2902,PVAAG-3027/3028,PVAAG-3091/3092,PVCA-17/18,PVCA-19/20,PVCA-37/38,PVCA-425/426,PVCA-7/8,PVCA-811/812,PVCA-863/864,PVCAG-2517/2518,PVCAG-2615/2616,PVGA-1091/1092,PVGA-1135/1136,PVGA-1153/1154,PVGA-1301/1302,PVGA-1351/1352,PVGA-1405/1406,PVGA-1513/1514,PVGA-1605/1606,PVGA-1611/1612,PVGA-1663/1664,PVGA-1843/1844,PVGA-1941/1942,PVGA-2083/2084,Sorghum-Xtxp46,sww-125,sww-166,sww-1678,sww-170,sww-1812,sww-2031,sww-2097,sww-2113,sww-2221,sww-2234,sww-2285,sww-2292,sww-2364,sww-2377,sww-2381,sww-2446,sww-2561,sww-364,sww-387,sww-463,sww-466,sww-564,sww-585,sww-651	**62**
Grand total		**319**
