# Supplementary figures and images for: A High-Density Simple Sequence Repeat-Based Genetic Linkage Map of Switchgrass
Source: G3 (Bethesda). 2012 Mar 1;2(3):357–70. doi: 10.1534/g3.111.001503 (PMC3291506; doi:10.1534/g3.111.001503)

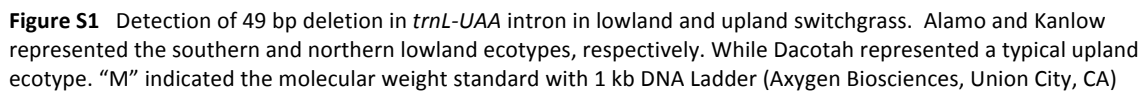

Supplement: Supporting Information [file supp_2.3.357_FigureS1.pdf]
