# Supplementary material for: A High-Density Simple Sequence Repeat-Based Genetic Linkage Map of Switchgrass
Source: G3 (Bethesda). 2012 Mar 1;2(3):357–70. doi: 10.1534/g3.111.001503 (PMC3291506; doi:10.1534/g3.111.001503)
Supplement: Supporting Information [file supp_2.3.357_FigureS2.pdf]

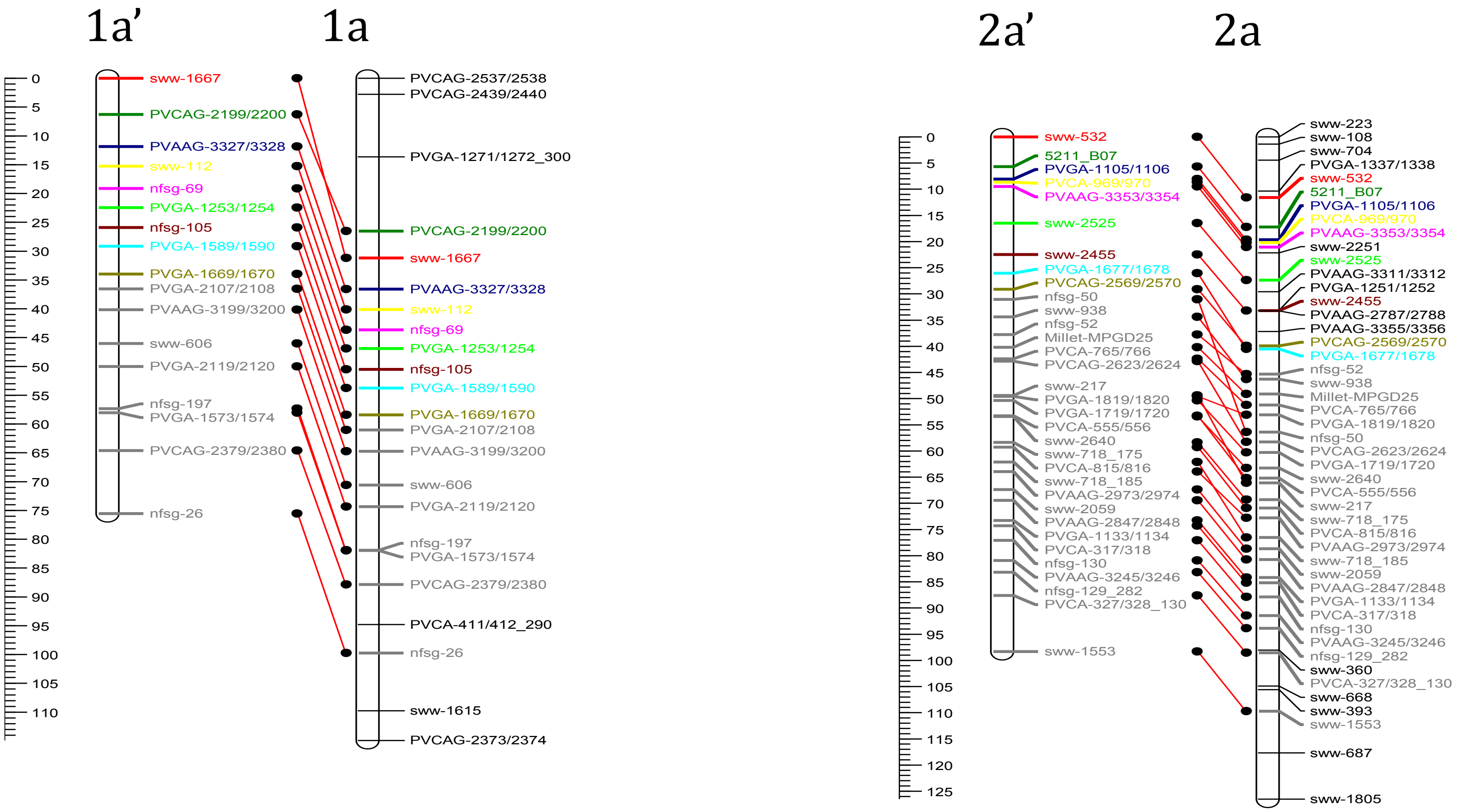

Figure S2 Collinerity between the initial and final framework linkage maps. Here only linkage groups (LGs) 1a and 2a are shown as the representatives of all LGs. The LG number of initial framework map is indicated by a quote symbol. All loci mapped with the same markers across LGs are highlighted with matching colors
