# Supplementary material for: A High-Density Simple Sequence Repeat-Based Genetic Linkage Map of Switchgrass
Source: G3 (Bethesda). 2012 Mar 1;2(3):357–70. doi: 10.1534/g3.111.001503 (PMC3291506; doi:10.1534/g3.111.001503)
Supplement: Supporting Information [file supp_2.3.357_FigureS3.pdf]

Figure S3

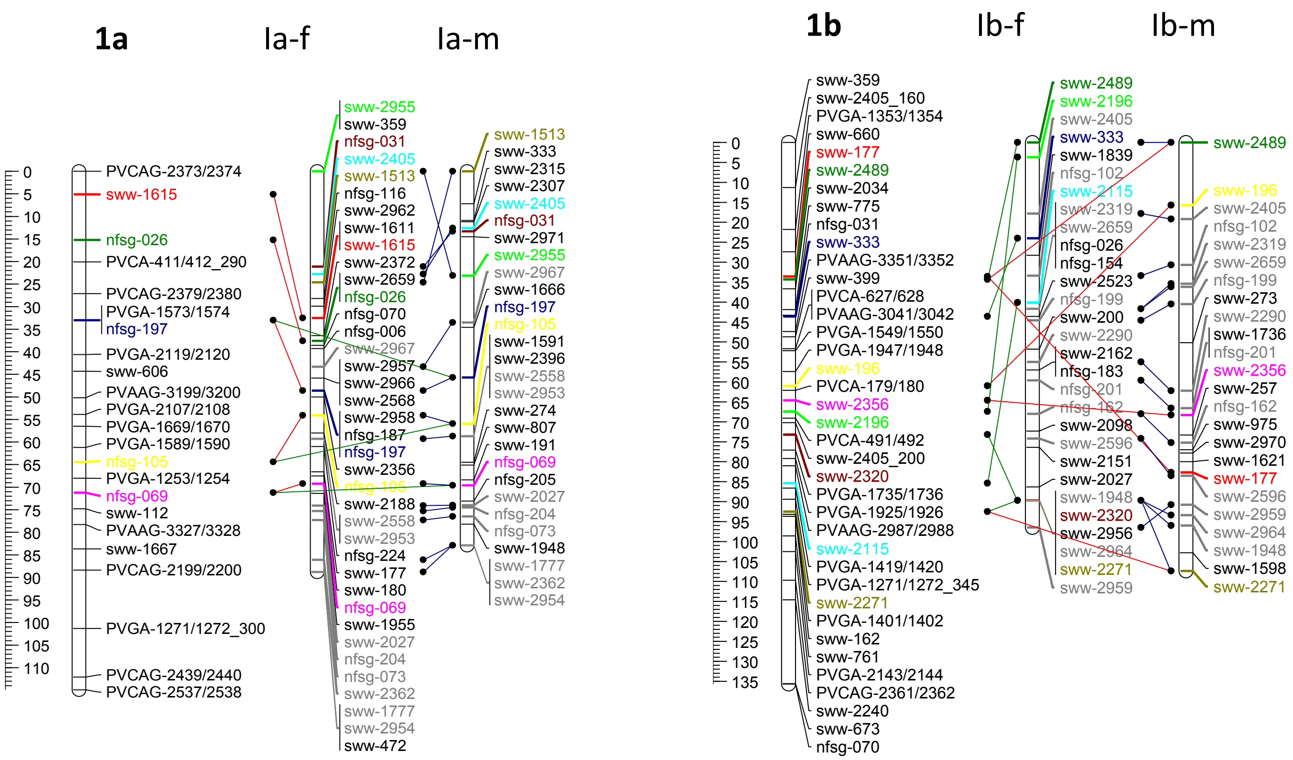
1a
Ia-f
Ia-m
PVCAG-2373/2374
sww-1615
nfsg-026
PVCA-411/412_290
PVCAG-2379/2380
PVGA-1573/1574
nfsg-197
PVGA-2119/2120
sww-606
PVAAG-3199/3200
PVGA-2107/2108
PVGA-1669/1670
PVGA-1589/1590
nfsg-105
PVGA-1253/1254
nfsg-069
sww-112
PVAAG-3327/3328
sww-1667
PVCAG-2199/2200
PVGA-1271/1272_300
PVCAG-2439/2440
PVCAG-2537/2538
sww-2955
sww-359
nfsg-031
sww-2405
sww-1513
nfsg-116
sww-2962
sww-1611
sww-1615
sww-2372
sww-2659
nfsg-026
nfsg-070
nfsg-006
sww-2967
sww-2957
sww-2966
sww-2568
sww-2958
nfsg-187
nfsg-197
sww-2356
nfsg-105
sww-2188
sww-2558
sww-2953
nfsg-224
sww-177
sww-180
nfsg-069
sww-1955
sww-2027
nfsg-204
nfsg-073
sww-2362
sww-1777
sww-2954
sww-472
sww-1513
sww-333
sww-2315
sww-2307
sww-2405
nfsg-031
sww-2971
sww-2955
sww-2967
sww-1666
nfsg-197
nfsg-105
sww-1591
sww-2396
sww-2558
sww-2953
sww-274
sww-807
sww-191
nfsg-069
nfsg-205
sww-2027
nfsg-204
nfsg-073
sww-1948
sww-1777
sww-2362
sww-2954
1b
Ib-f
Ib-m
sww-359
sww-2405_160
PVGA-1353/1354
sww-660
sww-177
sww-2489
sww-2034
sww-775
nfsg-031
sww-333
PVAAG-3351/3352
sww-399
PVCA-627/628
PVAAG-3041/3042
PVGA-1549/1550
PVGA-1947/1948
sww-196
PVCA-179/180
sww-2356
sww-2196
PVCA-491/492
sww-2405_200
sww-2320
PVGA-1735/1736
PVGA-1925/1926
PVAAG-2987/2988
sww-2115
PVGA-1419/1420
PVGA-1271/1272_345
sww-2271
PVGA-1401/1402
sww-162
sww-761
PVGA-2143/2144
PVCAG-2361/2362
sww-2240
sww-673
nfsg-070
sww-2489
sww-2196
sww-2405
sww-333
sww-1839
nfsg-102
sww-2115
sww-2319
sww-2659
nfsg-026
nfsg-154
sww-2523
nfsg-199
sww-200
sww-2290
sww-2162
nfsg-183
nfsg-201
nfsg-162
sww-2098
sww-2596
sww-2151
sww-2027
sww-1948
sww-2320
sww-2956
sww-2964
sww-2271
sww-2959
sww-2489
sww-196
sww-2405
nfsg-102
sww-2319
sww-2659
nfsg-199
sww-273
sww-2290
sww-1736
nfsg-201
sww-2356
sww-257
nfsg-162
sww-975
sww-2970
sww-1621
sww-177
sww-2596
sww-2959
sww-2964
sww-1948
sww-1598
sww-2271

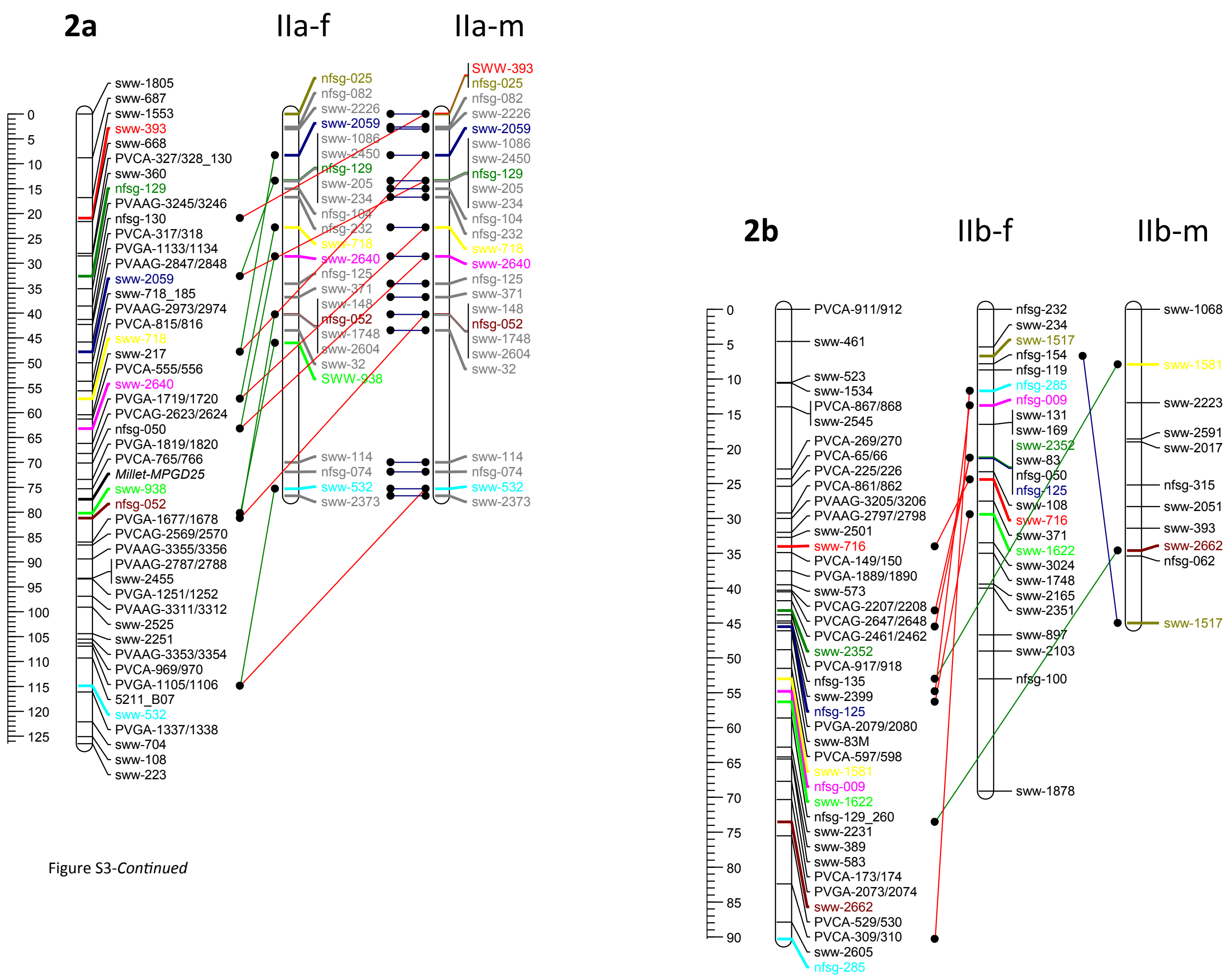

Figure S3-*Continued*

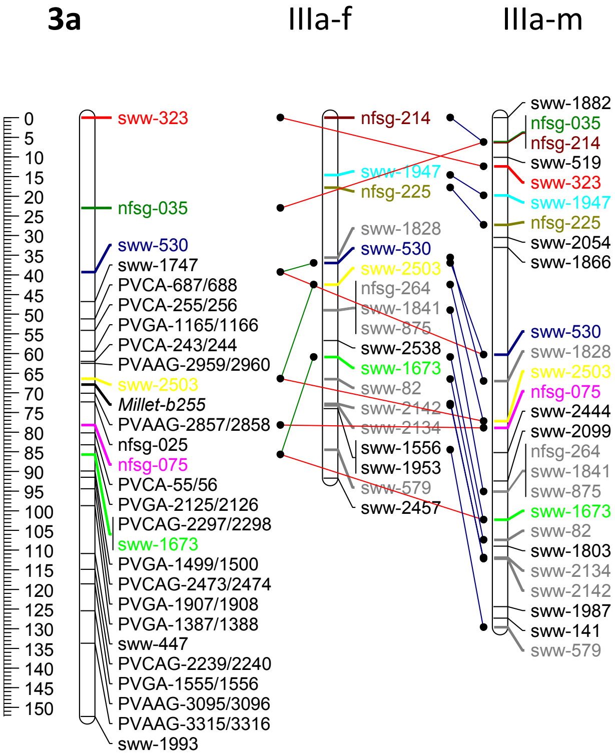

Figure S3-*Continued*

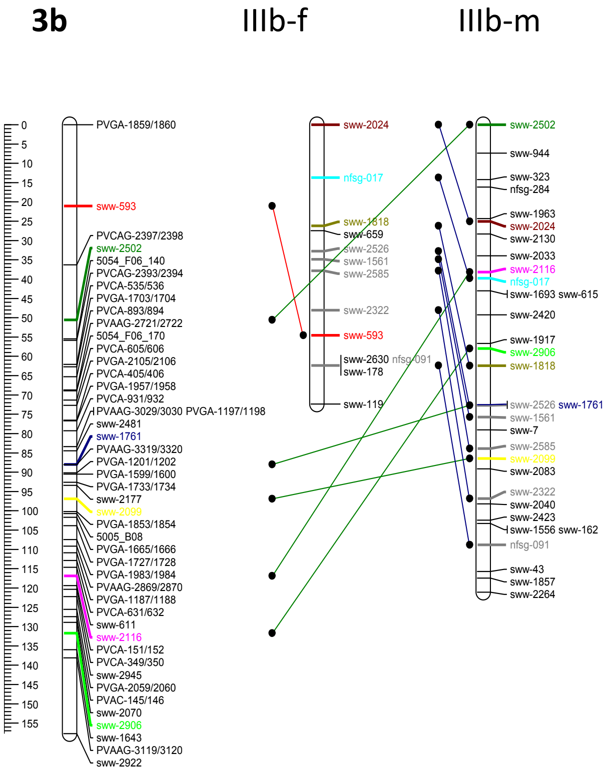

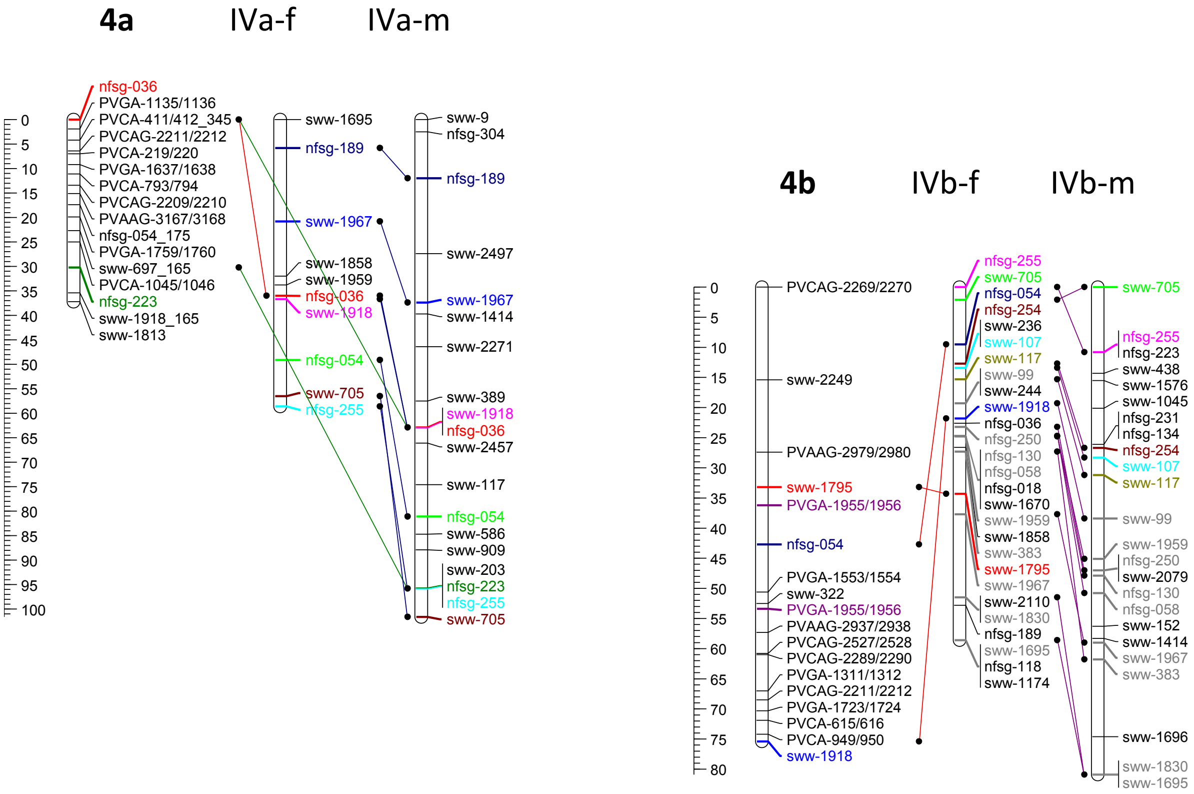

Figure S3-*Continued*

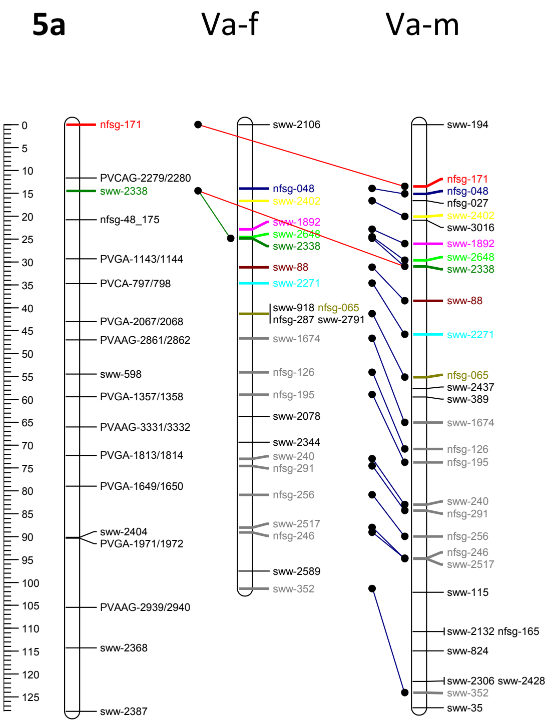

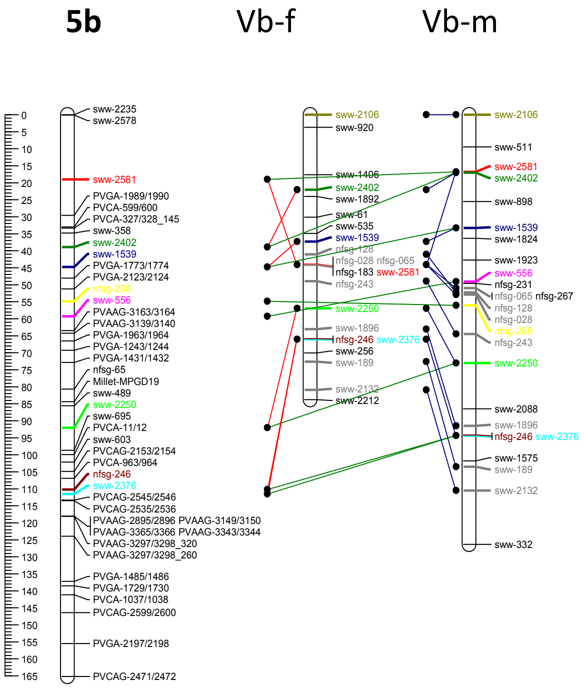

Figure S3-*Continued*

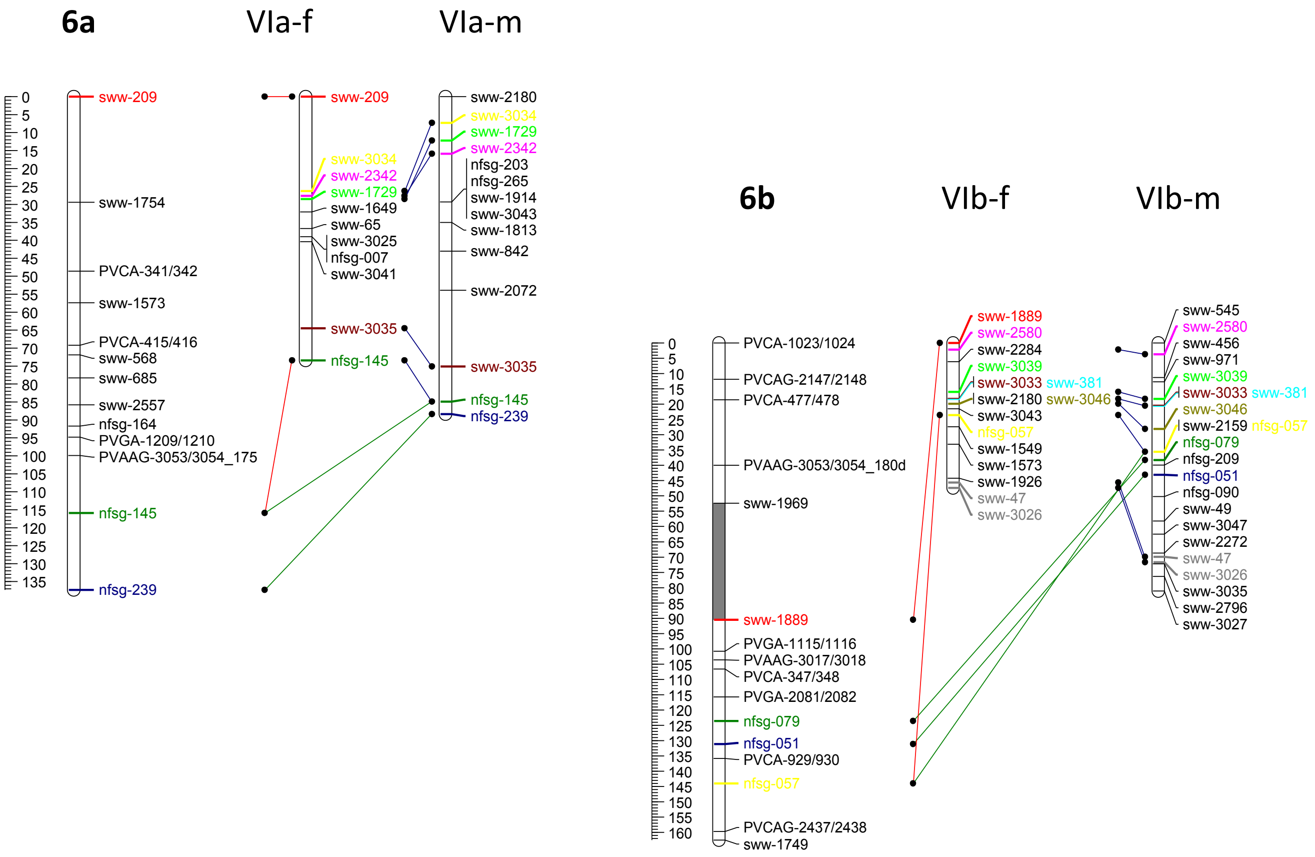

Figure S3-*Continued*

7a

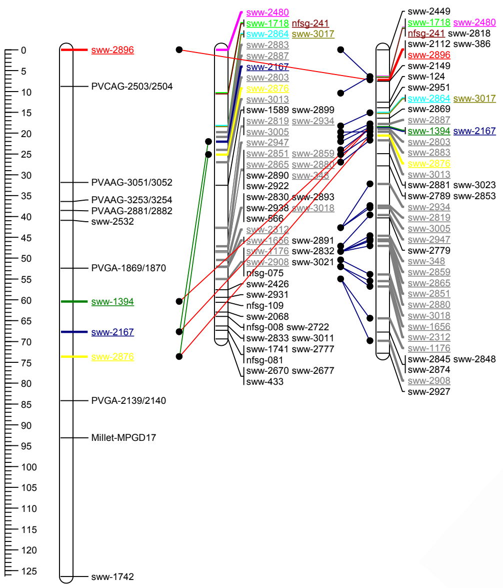

Figure S3-*Continued*

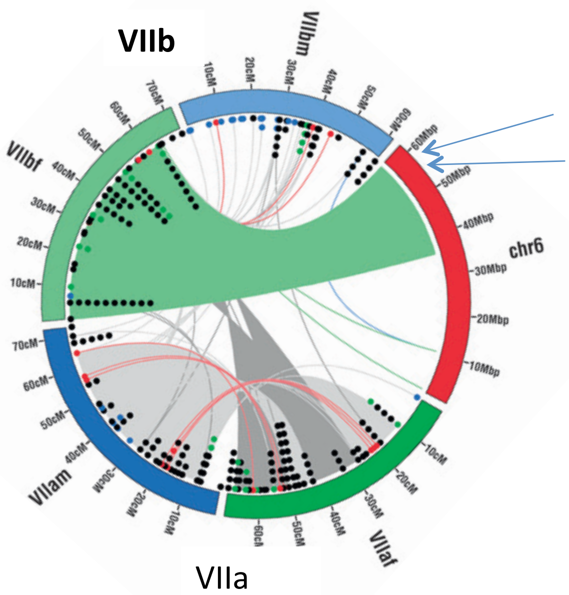

7b

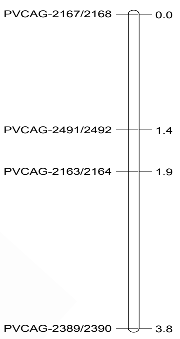

Circos image was taken from Okada *et al*. 2010 Genetics. Doi:10.1534/genetics. 110.113910

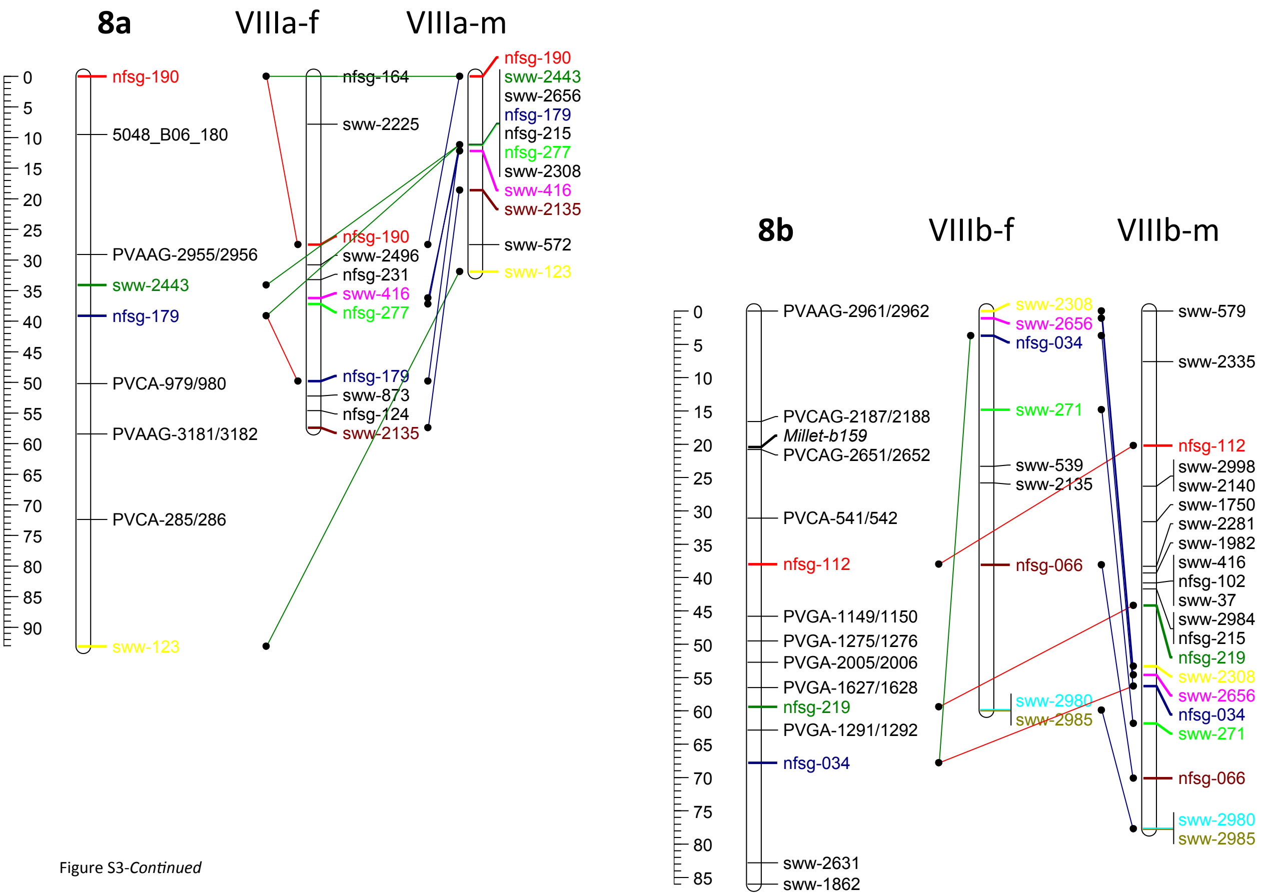

Figure S3-*Continued*

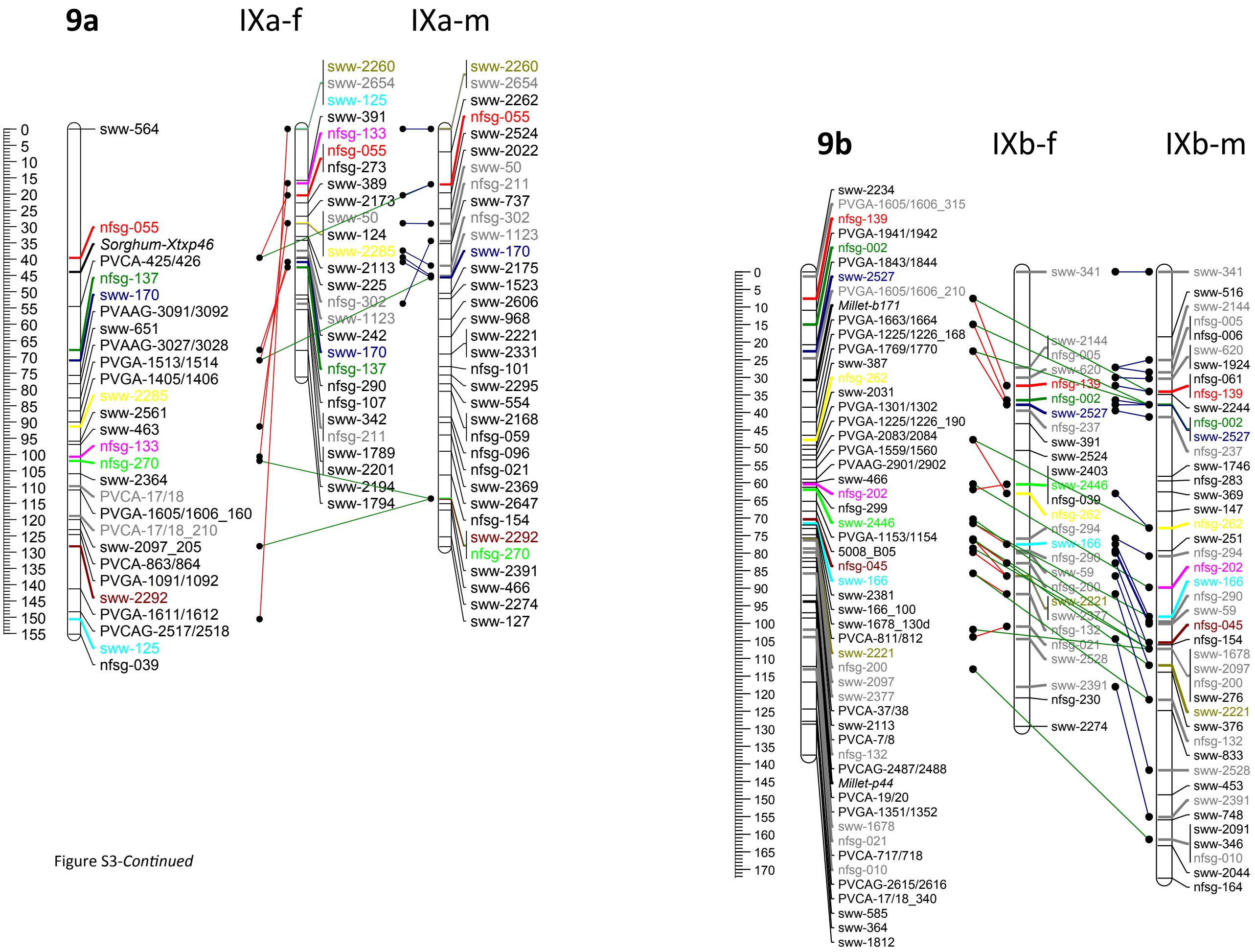

Figure S3-*Continued*

Figure S3 Collinerity between this and published linkage map (Okada *et al.* 2010). The names of linkage groups (LGs) in this study are indicated with Arabic numbers in bold, and the designation of LGs and two subgenomes ('a' and 'b') is consistent with the reference map (Okada *et al.* 2010). The letter 'f' and 'm' at the end of reference LGs (Roman number) denotes female and male parent maps, respectively. To compare the two maps, the duplicated loci amplified from same markers in reference map and the band sizes were removed. All loci mapped with the same markers across LGs are highlighted with matching colors.
