# Supplementary material for: A High-Density Simple Sequence Repeat-Based Genetic Linkage Map of Switchgrass
Source: G3 (Bethesda). 2012 Mar 1;2(3):357–70. doi: 10.1534/g3.111.001503 (PMC3291506; doi:10.1534/g3.111.001503)
Supplement: Supporting Information [file supp_2.3.357_TableS1.pdf]

Table S1 Expected progeny phenotypes and segregation ratios for disomic and tetrasomic inheritance in a selfed population of a tetraploid switchgrass plant

Inheritance mode	Marker scoring types	Genotypes of two bands observed [a]	Theoretical genotypes of selfed progeny [b]	Expected selfed progeny band pattern	Expected progeny ratio
Disomic	co-dominant	*AB*	*1AA, 2AB, 1BB*	a:ab:b	1:2:1
Tetrasomic		*ABBB*	*(1AABB,2ABBB),1BBBB*	ab:b	3:1
		AABB	*1AAAA,*(*8AAAB,18AABB,8ABBB*)*,1BBBB*	a:ab:b	1:34:1
		AAAB	*1AAAA,(2AAAB,1AABB)*	a:ab	1:3
Disomic	dominant	*A_*	(*1AA, 2A_*), *1_ _*	presence:absence	3:1
Tetrasomic		*A_ _ _*	*(1AA_ _ ,2A_ _ _),1_ _ _ _*	presence:absence	3:1
		AA_ _	*(1AAAA,8AAA_ ,18AA_ _ ,8A_ _ _),1_ _ _ _*	presence:absence	35:1
		*AAA_*	(1AAAA,2AAA_ ,1AA_ _)	presence:absence	all presence

[a] Italicized capital letters represent genotypes.
[b] The genotypes in parenthesis produce the same gel banding phenotypes.
