# Supplementary material for: A High-Density Simple Sequence Repeat-Based Genetic Linkage Map of Switchgrass
Source: G3 (Bethesda). 2012 Mar 1;2(3):357–70. doi: 10.1534/g3.111.001503 (PMC3291506; doi:10.1534/g3.111.001503)
Supplement: Supporting Information [file supp_2.3.357_TableS2.pdf]

Table S2 Detection of redundancy among published primers (Tobias *et al.* 2006, 2008; Wang *et al.* 2011) using bl2seq basic local alignment search tool (BLAST) (NCBI, Bethesda, MD)

Marker ID of Wang et al. (2011)	Marker ID of Okada et al. (2010)	SSR type	% Read (similarity)	Read length (bp)	E value	Bit score [b]
PVGA-1225/1226	NFSG005	gSSR	100	21	9.00E-08	39.9
PVGA-1901/1902	NFSG006	gSSR	100	22	6.00E-08	41.7
PVGA-1643/1644	NFSG066	gSSR	100	21	4.00E-09	39.9
PVGA-1419/1420	NFSG102	gSSR	100	20	2.00E-10	38.1
PVGA2-1329/1330	NFSG136	gSSR	100	20	7.00E-09	38.1
	NFSG249	gSSR	100	20	5.00E-08	39.9
PVGA-1669/1670	NFSG187	gSSR	100	21	4.00E-08	39.9
PVGA-1627/1628	NFSG215	gSSR	100	21	2.00E-09	38.9
PVGA-1657/1658	NFSG243	gSSR	100	21	1.00E-08	30.7
PVGA-1867/1868	SWW2864	eSSR	100	20	2.30E-08	39.9
PVGA-2035/2036	SWW2881	eSSR	100	21	4.00E-08	39.9
PVGA-1295/1296	SWW2954	eSSR	100	21	7.00E-09	38.8
PVCAG-2245/2246	SWW3033	eSSR	100	21	2.10E-09	30.7
PVGA6-1793/1794	SWW2839	eSSR	76	16	4.00E-06	38.8
PVGA5-1697/1698	sww2845	eSSR	100	21	1.41E-09	38.8
PVCA- 985/986	SWW3043	eSSR	100	18	8.10E-07	38.1
sww1 [a]	4821_A04	eSSR	100	20	2.00E-09	38.1
sww842	4934_H04	eSSR	100	20	1.00E-09	38.1
SWW35	No reverse sequence hit in GenBank	eSSR				
NFSG028	Redundant with NFSG027	gSSR				

[a] The last four primer pairs are the comparison of primer pairs from same research group (Tobias *et al.* 2006, 2008; Okada *et al.* 2010)

[b] *Note* the bit score represents the normalized value that can be used to compare alignment scores from different searches
