# Supplementary material for: A High-Density Simple Sequence Repeat-Based Genetic Linkage Map of Switchgrass
Source: G3 (Bethesda). 2012 Mar 1;2(3):357–70. doi: 10.1534/g3.111.001503 (PMC3291506; doi:10.1534/g3.111.001503)
Supplement: Supporting Information [file supp_2.3.357_TableS3.pdf]

Table S3 Presentation of identical bridge markers in the linkage groups (LGs) of this map and a previous study (Okada *et al*. 2010)

LGs in this study	Corresponding LGs and subgenome designation of Okada *et al*. 2010	No. of bridge markers	LGs and alternate subgenome designation of Okada *et al*. 2010	No. of bridge markers
1a	Ia	5	Ib	0
1b	Ib	10	Ia	5
2a	IIa	8	IIb	2
2b	IIb	8	IIa	1
3a	IIIa	6	IIIb	1
3b	IIIb	7	IIIa	0
4a[a]	IVa	4	IVb	1
4b	IVb	3	IVa	2
5a	Va	3	Vb	0
5b	Vb	8	Va	2
6a	VIa	3	VIb	1
6b	VIb	4	Via	0
7a	VIIa	4	VIIb	0
7b	VIIb	0	VIIa	0
8a	VIIIa	4	VIIIb	0
8b	VIIIb	3	VIIIa	0
9a	IXa	8	IXb	1
9b	IXb	16	Ixa	2
Total		**102**		**15**

[a] Two bridge markers (nfsg-054 and sww-1918) were shared by both LG 4a and 4b.
