# Supplementary material for: A High-Density Simple Sequence Repeat-Based Genetic Linkage Map of Switchgrass
Source: G3 (Bethesda). 2012 Mar 1;2(3):357–70. doi: 10.1534/g3.111.001503 (PMC3291506; doi:10.1534/g3.111.001503)
Supplement: Supporting Information [file supp_2.3.357_TableS4.pdf]

Table S4 A list of switchgrass markers and their corresponding nine chromosomes of foxtail millet by BlastN analysis in Phytozome v7.0

Chr. of foxtail millet	Switchgrass markers	Total
I	nfsg-026,nfsg-105,nfsg-197,PVAAG-2987/2988,PVAAG-3327/3328,PVAAG-3351/3352,PVCA-179/180,PVCA-411/412,PVCA-627/628,PVCAG-2199/2200,PVCAG-2379/2380,PVCAG-2439/2440,PVCAG-2537/2538,PVGA-1253/1254,PVGA-1271/1272,PVGA-1353/1354,PVGA-1573/1574,PVGA-1669/1670,PVGA-1735/1736,PVGA-1925/1926,PVGA-1947/1948,PVGA-2119/2120,sww-112,sww-1615,sww-162,sww-1667,sww-177,sww-196,sww-2115,sww-2196,sww-2240,sww-2271,sww-2320,sww-2405,sww-2489,sww-333,sww-359,sww-606,sww-660,sww-673	**40**
II	PVCA-529/530,5211_B07,Millet-MPGD25,nfsg-050,nfsg-052,nfsg-125,nfsg-129,nfsg-130,nfsg-285,PVAAG-2787/2788,PVAAG-2797/2798,PVAAG-2847/2848,PVAAG-3205/3206,PVAAG-3245/3246,PVAAG-3311/3312,PVAAG-3353/3354,PVAAG-3355/3356,PVCA-173/174,PVCA-269/270,PVCA-309/310,PVCA-317/318,PVCA-327/328,PVCA-555/556,PVCA-597/598,PVCA-765/766,PVCA-815/816,PVCA-861/862,PVCA-867/868,PVCA-911/912,PVCA-917/918,PVCA-969/970,PVCAG-2207/2208,PVCAG-2461/2462,PVCAG-2569/2570,PVCAG-2623/2624,PVGA-1105/1106,PVGA-1133/1134,PVGA-1337/1338,PVGA-1677/1678,PVGA-1719/1720,PVGA-1819/1820,PVGA-2079/2080,sww-108,sww-1553,sww-1581,sww-1622,sww-1805,sww-217,sww-223,sww-2231,sww-2251,sww-2352,sww-2455,sww-2501,sww-2525,sww-2545,sww-2640,sww-2662,sww-360,sww-389,sww-393,sww-461,sww-523,sww-532,sww-573,sww-583,sww-668,sww-704,sww-716,sww-718,sww-938	**71**
III	5005_B08,5054_F06,Millet-b255,nfsg-025,nfsg-035,nfsg-075,PVAAG-2721/2722,PVAAG-2869/2870,PVAAG-2959/2960,PVAAG-3095/3096,PVAAG-3119/3120,PVAAG-3315/3316,PVAAG-3319/3320,PVCA-151/152,PVCA-405/406,PVCA-55/56,PVCA-631/632,PVCA-687/688,PVCA-893/894,PVCA-931/932,PVCAG-2239/2240,PVCAG-2297/2298,PVCAG-2393/2394,PVCAG-2473/2474,PVGA-1115/1116,PVGA-1187/1188,PVGA-1197/1198,PVGA-1201/1202,PVGA-1665/1666,PVGA-1703/1704,PVGA-1727/1728,PVGA-1957/1958,PVGA-1983/1984,PVGA-2059/2060,PVGA-2105/2106,PVGA-2125/2126,sww-1643,sww-1673,sww-1761,sww-1993,sww-2070,sww-2177,sww-2481,sww-2502,sww-2503,sww-2906,sww-2922,sww-2945,sww-323,sww-447,sww-530,sww-593,sww-611	**53**
IV	PVCA-1045/1046,nfsg-036,nfsg-054,PVAAG-3167/3168,PVCA-219/220,PVCA-615/616,PVCA-793/794,PVCA-949/950,PVCAG-2269/2270,PVCAG-2527/2528,PVGA-1311/1312,PVGA-1553/1554,PVGA-1637/1638,PVGA-1723/1724,PVGA-1759/1760,PVGA-1955/1956,sww-1795,sww-697	**18**
V	PVCA-1037/1038,Millet-MPGD19,nfsg-048,nfsg-065,nfsg-246,PVAAG-2861/2862,PVAAG-2895/2896,PVAAG-2939/2940,PVAAG-3139/3140,PVAAG-3149/3150,PVAAG-3331/3332,PVCA-11/12,PVCA-797/798,PVCAG-2153/2154,PVCAG-2197/2198,PVCAG-2279/2280,PVCAG-2471/2472,PVCAG-2535/2536,PVCAG-2545/2546,PVGA-1143/1144,PVGA-1243/1244,PVGA-1963/1964,PVGA-1989/1990,PVGA-2123/2124,sww-1539,sww-2235,sww-2338,sww-2368,sww-2376,sww-2387,sww-2402,sww-2404,sww-2578,sww-2581,sww-358,sww-598,sww-603	**37**
VI	PVCA-1023/1024,nfsg-145,nfsg-164,nfsg-239,PVAAG-3017/3018,PVAAG-3053/3054,PVCA-341/342,PVGA-1209/1210,sww-1754,sww-1889,sww-1969,sww-209,sww-2557,sww-568,sww-685	**16**
VII	Millet-MPGD17,PVCAG-2163/2164,PVCAG-2389/2390,PVCAG-2503/2504,PVGA-1869/1870,sww-1394,sww-1742,sww-2532	**8**
VIII	5048_B06,nfsg-034,nfsg-112,nfsg-219,PVAAG-2961/2962,PVAAG-3181/3182,PVCA-541/542,PVCAG-2187/2188,PVGA-1149/1150,PVGA-1275/1276,PVGA-1627/1628,sww-123,sww-1862,sww-2443	**14**
IX	5008_B05,Millet-b171,Millet-p44,nfsg-010,nfsg-039,nfsg-055,nfsg-132,nfsg-133,nfsg-200,nfsg-202,nfsg-262,nfsg-270,PVAAG-2901/2902,PVAAG-3027/3028,PVAAG-3091/3092,PVCA-17/18,PVCA-19/20,PVCA-37/38,PVCA-425/426,PVCA-7/8,PVCA-811/812,PVCA-863/864,PVCAG-2517/2518,PVCAG-2615/2616,PVGA-1091/1092,PVGA-1135/1136,PVGA-1153/1154,PVGA-1301/1302,PVGA-1351/1352,PVGA-1405/1406,PVGA-1513/1514,PVGA-1605/1606,PVGA-1611/1612,PVGA-1663/1664,PVGA-1843/1844,PVGA-1941/1942,PVGA-2083/2084,Sorghum-Xtxp46,sww-125,sww-166,sww-1678,sww-170,sww-1812,sww-2031,sww-2097,sww-2113,sww-2221,sww-2234,sww-2285,sww-2292,sww-2364,sww-2377,sww-2381,sww-2446,sww-2561,sww-364,sww-387,sww-463,sww-466,sww-564,sww-585,sww-651	**62**
Grand total		**319**
